# Green silver nanoparticles of *Khaya senegalensis* as dual inhibitors of viral thymidine kinase and 3 C protease: metabolomics, and computational insights

**DOI:** 10.1038/s41598-026-43691-6

**Published:** 2026-03-29

**Authors:** Heba A. El Gizawy, Rehab H. Abd El-Aleam, Nevine H. Hassan

**Affiliations:** 1 Department of Pharmacognosy, Faculty of Pharmacy, October 6 University, 6th of October City, Giza, 12585 Egypt; 2 Pharmaceutical Chemistry Department, Faculty of Pharmacy, Modern University for Technology and Information MTI, Cairo, 11571 Egypt; 3 Department of Pharmacognosy, Faculty of Pharmacy, Modern University for Technology and Information, Cairo, 11571 Egypt

**Keywords:** *Khaya senegalensis*, UPLC/T-TOF–MS/MS, Antiviral, Silver nanoparticles, Docking study, Molecular dynamics, ADME, Biochemistry, Biological techniques, Biotechnology, Chemistry, Drug discovery, Microbiology

## Abstract

To the best of our knowledge, this is the first study to evaluate green synthesized silver nanoparticles loaded *Khaya senegalensis* (Desr.) A. Juss. against HSV-1 and Coxsackie B4, while providing mechanistic insights through enzyme inhibition and in silico analyses. FTIR, HRTEM, UV–visible spectroscopy, particle size, and zeta potential analyses further characterized the nanoparticles, which exhibited colloidal stability (− 20.7 ± 5.26 mV), predominantly spherical morphology, and a core size of 8–38 nm by HRTEM. The larger hydrodynamic diameter observed by DLS (~ 463 nm) reflects the core plus capping biomolecules and solvation layer, consistent with effective nanoparticle stabilization. KS-AgNPs leaves showed the strongest antiviral effect against HSV-1 and Coxsackie B4, with an IC₅₀ of 99.65 ± 1.84 µg/mL. This was better than the crude leaf extract (116.26 ± 1.28 µg/mL) but less potent than the standard drug acyclovir (79.25 ± 0.14 µg/mL). Metabolomic profiling identified thirty secondary metabolites, with molecular docking and dynamics highlighting myricetin as a key antiviral compound (binding energies: −10.98 kcal/mol for thymidine kinase, − 9.42 kcal/mol for 3 C protease). Furthermore, pharmacokinetics, and ADME studies for myricetin was performed. In vitro enzyme inhibition assays confirmed suppression of thymidine kinase (IC₅₀ = 0.249 ± 0.007 µg/mL) and 3 C protease (IC₅₀ = 0.732 ± 0.028 µg/mL). The results indicate that biogenic KS-AgNPs have notable antiviral effects, and that metabolites from *Khaya senegalensis* may serve as potential candidates for future antiviral therapies.

**Supplementary Information:**

The online version contains supplementary material available at 10.1038/s41598-026-43691-6.

## Introduction

Plants are the key source for drug discovery and novel healthcare products^1,2^. *Khaya senegalensis* (*K. senegalensis*) leaves belongs to family Meliaceae, and also called African or dry zone mahogany, it is a medicinal and timber tree native to the savannahs of sub-Saharan Africa, including Senegal, Nigeria, Ghana, Uganda, and Cameroon^3^. *Khaya senegalensis* has been found to produce a variety of secondary metabolites like tannins, steroids, flavonoids, saponins, alkaloids, glycosides, anthocyanin, anthraquinones, terpenoids, Fatty acids and Limonoids^4–6^. The plant also displays a wide array of biological activities including antiplasmodial^7^, antimalarial^8^, anthelmintic^9^, anticancer^10^, antifeedant^11^, antitrypanosomal^12^, antioxidant^13^, anti-inflammatory^14^ wound-healing^4^, and antinociceptive^15^ activities.

In recent decades, the effectiveness of antiviral agents has been declined due to the emergence of novel resistance mechanisms in viruses, leading to multi-resistant and, in some cases, highly resilient viral strains^16^. Natural medicines with minimal side effects are being explored to address infections worsened by drug misuse^17^.

Herpes simplex virus type I (HSV-1) a common human infection with no seasonal pattern, affecting 70–80% of people by adulthood^18^. Painful blisters or ulcers can appear on the lips, eyes, oral mucous membranes, and genital area^19^.

Coxsackie B4 (CoxB4) is associated with a wide range of diseases worldwide and can cause aseptic meningitis, encephalitis, myocarditis, and pericarditis^20^. CoxB4 infection can lead to adolescent-onset insulin-dependent diabetes mellitus, its most serious chronic complication^21^. Ongoing research is needed to find safe, affordable antiviral agents that can effectively combat these viruses^22^.

Nanotechnology is regarded as a key and highly significant field in materials science^23^. Nanoscale materials possess enhanced physicochemical properties due to their high surface-area-to-volume ratio^24^. Silver nanoparticles (Ag-NPs) can enter human cells, raising concerns about cytotoxicity and long-term health effects, and their release into the environment may negatively impact aquatic ecosystems^25^. Green synthesis of nanoparticles using environmentally friendly substances as an emerging branch in nanotechnology^26^. Green-synthesized silver nanoparticles (AgNPs) offer a promising alternative, and recent studies highlight their antiviral potential^27,28^. Viral diseases continue to rise, and overuse of antiviral drugs may drive resistance^29^.

**Scheme 1:**
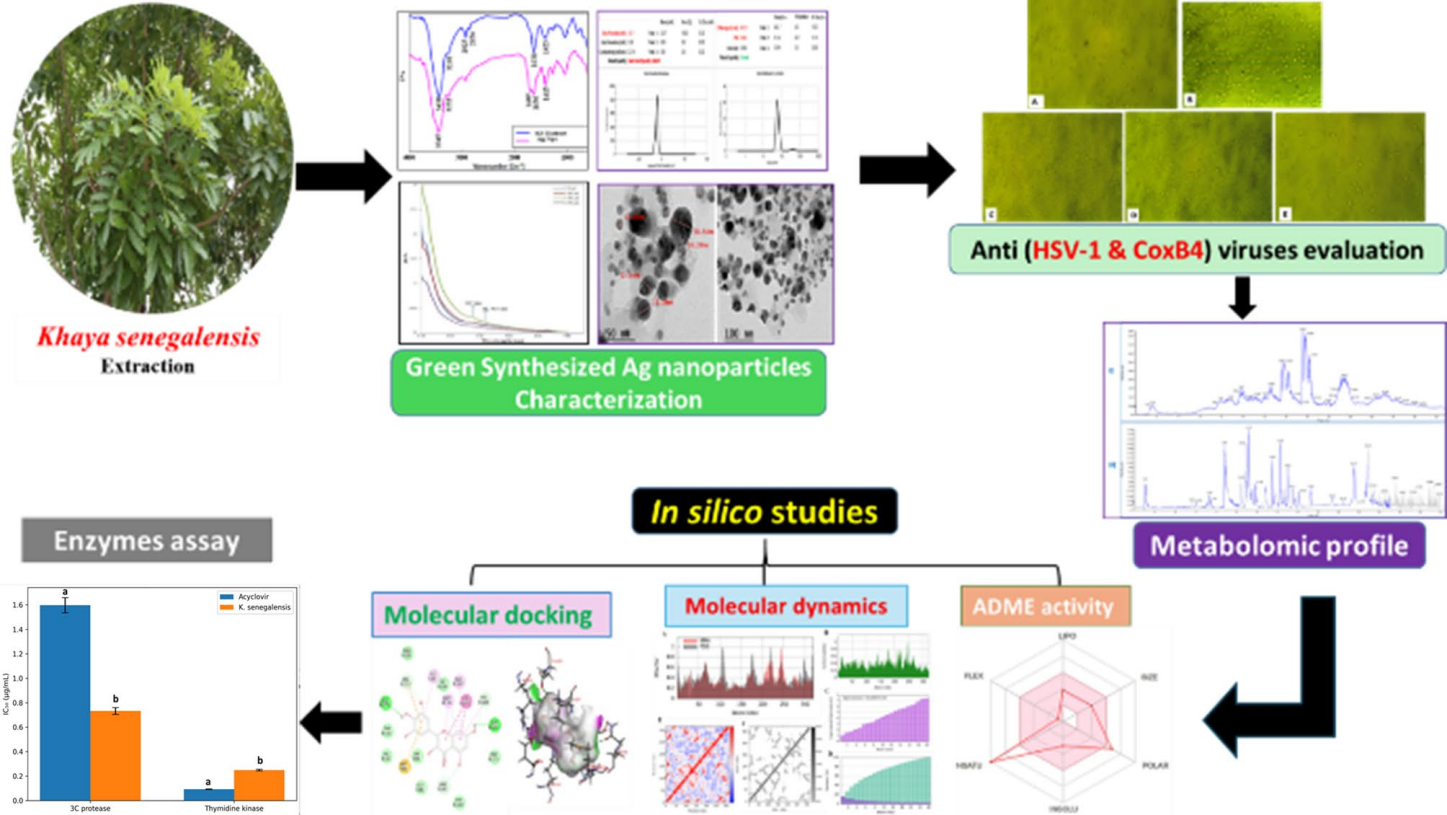
Overview of Ag nanoparticles (KS-AgNPs) characterization, in vitro antiviral inhibition assay, metabolomics profiling of *K. senegalensi*s leaves, molecular docking study, molecular dynamics simulations, ADME analysis, and in-vitro enzymatic activity assessment.

Despite extensive studies on silver nanoparticles for antimicrobial use, there is limited research on green-synthesized AgNPs from *Khaya senegalensis* leaves, and their antiviral activity remains largely unexplored although; it is rich in bioactive secondary metabolites, including limonoids, flavonoids, and phenolics, which are known for their antiviral and immunomodulatory properties. Moreover, the specific interactions of *K. senegalensis* biomolecules with viral enzymes and their mechanisms of action have not been studied. This work is the first to report the in vitro antiviral potential of green-synthesized AgNPs from *K. senegalensis*, supported by metabolomic profiling using [UPLC/T-TOF–MS/MS, molecular docking, Pharmacokinetic profiling, ADME (absorption, distribution, metabolism, and excretion)] predictions of the highest-affinity compound, and molecular dynamics simulations. Finally, enzyme inhibition assays confirmed the mechanism of action, showing that the extract effectively suppresses the targeted viral enzymes and highlighting its potential as a novel antiviral nanotherapeutics, as illustrated in Scheme 1.

## Materials and methods

### Chemicals

Silver nitrate (AgNO₃) was procured from E-Merck (Darmstadt, Germany), while HPLC-grade methanol was obtained from SD Fine-Chem Limited (Mumbai, India).

### Plant collection

Plant leaves were gathered from El Zohria Garden in Giza, Egypt, in January 2023. Species identification was authenticated by Dr. Ehsan El Sayed, Executive Manager of the garden. With the permission of the garden manger, that research does not affect plant also having moral responsibility to ensure that our research does not harm species’ survival. A voucher specimen was archived in the herbarium of the Pharmacognosy Department, Faculty of Pharmacy, October 6 University, No. (Kh 2023/12).

#### Extraction and preparation

Leaves of the plant were air-dried, powdered (150 g) and extracted with methanol (3 L). The solvent was evaporated under reduced pressure (50 ᴼC) to yield a brown sticky residue 34 g (22.6%). Until examination, it was kept at − 5 °C.

### Synthesis of KS-silver nanoparticles (AgNPs)

AgNPs were synthesized at room temperature by reduction of 10 mL of a 2.5 mM AgNO_3_ solution with varying concentrations of *K. senegalensis (*KS) extract, ranging from 50 µL to 200 µL, derived from a stock solution containing 0.2 g of extract/ 10 mL of methanol. The mixture was gently agitated by hand and subsequently left to stand in the dark.

**Characterization of green synthesized KS-AgNPs**

#### Determination of particle size (PS), and zeta-potential (ZP)

Particle size was measured using dynamic light scattering (DLS); zeta potential was also assessed via electrophoretic mobility. Analyses used a Malvern Zetasizer Nano-ZS (Malvern, Worcestershire, United Kingdom) at 90°, in a 10 mm cuvette at 25 °C. Freshly prepared KS-AgNPs suspension (1 mL) was sonicated (Crest Ultrasonics, USA) for 10–15 min to ensure uniform dispersion^30^.

#### High resolution transmission electron microscopy (HRTEM)

KS-AgNPs morphology and particle dimensions were analyzed via HR-TEM (JEOL JEM-2100, 80 kV, Japan). A drop of sample was dried on a carbon-coated copper grid; excess was removed with filter paper. Negative staining with 1% phosphotungestic acid to improve imaging contrast^31^.

#### UV-visible spectroscopy

The formation of KS-AgNPs was observed by analyzing the UV-Vis spectrum of the reaction medium with a V-730 UV-Vis spectrophotometer (JASCO, Japan), covering a wavelength ( 200 to 800 nm) to detect the metal band wave length^32^.

#### Fourier transform infrared spectroscopy (FTIR)

For FTIR analysis, KS extract and KS-AgNPs were mixed with KBr and pressed into thin pellets. Spectra were recorded using a Nicolet Impact-400 FT-IR spectrophotometer (Jasco, Japan) across 400–4000 cm⁻¹ to characterize the functional groups responsible for reduction and stabilization during nanoparticle formation^33^.

### In-vitro antiviral activity

#### Virus, strains, and cell culture conditions

Herpes simplex virus type 1 (HSV-1) and Coxsackievirus B4 (CoxB4), were obtained from the Laboratory of Virology at the Science Way for Scientific Research and Consultations, Microbiology and Immunology Department, Faculty of Medicine for Girls, Al-Azhar University, Egypt. Vero cells (CCL-81), (ATCC) adherent kidney epithelial cells from Cercopithecus aethiops, (CCL-81) was cultured in RPMI 1640 medium (Gibco, Tunisia), supplemented with 10% fetal bovine serum, 2 mM L-glutamine, 100 U/mL penicillin, and 100 µg/mL streptomycin. The cells were incubated at 37 °C under humid conditions with 5% CO_2_ to promote optimal growth.

#### Determination of cytotoxicity on VERO cells

The maximum non-toxic concentration (MNTC) of KS extract and green-synthesized KS-AgNPs in Vero cells was assessed via MTT assay. After forming confluent monolayers in 96-well plates, cells were exposed to serial dilutions of each compound in minimum essential medium, with three wells left untreated. Plates were incubated at 37 °C and 5% CO₂ for 48 h, observing cytotoxic signs. MTT solution (20 µL, 5 mg/mL) was added, shaken at 150 rpm, and incubated for up to 5 h to form formazan crystals. After discarding the medium and drying, crystals were solubilized with 200 µL DMSO. Absorbance was measured at 560 nm, corrected at 620 nm to determine cell viability^34^.

#### Antiviral MTT assay protocol

Antiviral activity of KS extract and KS-AgNPs was assessed using an MTT assay. Virus-infected cells (10,000 per well) were seeded in 96-well plates with 200 µL medium per well. Sample dilutions were mixed 1:1 with virus suspension, incubated for 1 h, then 100 µL of the mixture was added to each well and gently shaken for 5 min. Three wells served as blank controls. Cells were allowed to adhere overnight at 37 °C with 5% CO₂, followed by a 24 h incubation for viral activity. Next, 20 µL of MTT solution (5 mg/mL in PBS) was added, and incubated 1–5 h for formazan formation, after which the medium was removed, and crystals were solubilized in 200 µL DMSO. Optical density was measured at 560 nm, with 620 nm background subtraction, to determine cell viability^28^.

The following equation was used to calculate the antiviral activity of four determinations:

$$\text{Antiviral activity} (\%)=\frac{(\text{Optical density of treated cells}-\text{Optical density of virus control})}{(\text{Optical density of cell control}-\text{Optical density of virus control})}\times 100.$$

### Metabolomics profiling study

A 50 mg methanolic leaves extract of *K. senegalensis* was prepared at the Proteomics and Metabolomics Department, Children’s Cancer Hospital Egypt 57,357, by suspending the sample in a solvent mixture of Milli-Q water, methanol, and acetonitrile (50:25:25). The mixture was sonicated for 10 min and centrifuged at 10,000 rpm for 10 min. A 50 µL supernatant was diluted to 1 mL and analyzed at 40 °C using the Exion LC–Triple TOF 5600+ (SCIEX, Framingham, MA, USA) at 40 °C. Chromatographic separation was performed on an X Select HSS T3 C18 column (2.1 × 150 mm, 2.5 μm; Waters) with a Phenomenex inline precolumn filter (0.5 μm × 3.0 mm).

Phytochemical profiling of *K. senegalensis* was performed on a UPLC–T-TOF–MS/MS system in both negative- and positive-ion modes by injecting 10 µL of a 1 µg/µL extract at 0.3 mL/min; in negative mode the mobile phase comprised solvent A (5 mM ammonium formate buffer, pH 8 adjusted with NaOH, containing 1% methanol) and solvent B (100% acetonitrile)^35^. In positive mode solvent A was 5 mM ammonium formate buffer, pH 3 adjusted with formic acid, containing 1% methanol, with solvent B unchanged; elution followed an initial isocratic hold at 90:10 (A: B) for 0–1 min, a linear gradient to 10:90 over 1.1–20.9 min, an isocratic hold at 10:90 for 21–25 min, and re-equilibration at 90:10 for 25.1–28 min; a 10 µL blank injection confirmed absence of carryover. Phytoconstituents were identified using Analyst TF 1.7.1, while data were processed with PeakView 2.2 (SCIEX, Framingham, MA, USA) and MS-DIAL 3.70^36^.

Mass spectra were acquired on an AB SCIEX Triple TOF 5600 + outfitted with a Duo-Spray electrospray ionization source, scanning from m/z 50 to 1 100. Candidate molecular formulas were generated with a mass accuracy cutoff of 10 ppm. Compound identities; were then confirmed by combining chromatographic retention times, MS² fragmentation patterns, database matching (e.g., METLIN, HMDB) and cross-referencing the primary literature^37^.

### In silico studies

#### Molecular docking

Docking was performed using ligand structures from single-crystal X-ray diffraction and protein models of HSV-1 thymidine kinase (PDB: 1KI2) and Coxsackie virus B4 3 C protease (PDB: 3ZZ5) from the RCSB Protein Data Bank. Protein preparation was done using Autodock Tools 1.5.7^38^. Open-Babel software was used for minimizing energy and Marvin-Sketch was used to sketch the compounds. The Autodock Vina 1.1.2 software was used for performing molecular docking studies^39,40^. Docking grids were centered on the active site to encompass the receptor’s helical exterior active site regions. Grid dimensions were set to 40 × 26 × 44 points for 1KI2 and 30 × 30 × 25 points for 3ZZ5, with a grid spacing of 1.0 Å^41^. Grid centers were set at coordinates (9.0, − 17.0, 16.0) for thymidine kinase and (17, 33, 38) for 3 C protease to ensure coverage of the entire binding pockets, including both the natural ligand binding pockets and active site surfaces. Final analysis and visualization were performed using Discovery Studio (Dassault Systèmes BIOVIA).

#### Molecular dynamics

The most stable binding conformations of myricetin with HSV-1 thymidine kinase and Coxsackie B4 virus 3 C protease, as identified through molecular docking, were selected for subsequent Normal Mode Analysis (NMA), a simplified molecular dynamics approach rather than full classical MD simulation. NMA was chosen because it provides rapid and computationally efficient insights into large-scale protein flexibility and equilibrium motion, making it suitable for preliminary screening of protein–ligand stability. These complexes were subjected to Normal Mode Analysis (NMA) using the iMODS web server to evaluate their intrinsic molecular flexibility and equilibrium motion within internal coordinates. NMA provided critical insights into the deformability, correlated atomic movements, and overall structural stability of the protein–ligand complexes. The analysis was conducted via the iMODS platform on July 22, 2025.

#### Pharmacokinetics and ADME prediction

Myricetin’s pharmacokinetic profile was assessed using SwissADME, a web-based tool developed by the Molecular Modeling Group at the Swiss Institute of Bioinformatics (available at http://www.swissadme.ch). SwissADME predicts key descriptors relevant to drug development, including lipophilicity (octanol–water partition coefficient), aqueous solubility, blood–brain barrier permeability, and gastrointestinal absorption (HIA), providing insight into the compound’s potential bioavailability and pharmacological performance^42^.

### In-vitro inhibition assay of thymidine kinase and 3 C protease enzymes

The methanolic leaves extract of *K. senegalensis* was tested at the VACSERA Confirmatory Diagnostic Unit in Cairo, Egypt, for its ability to inhibit Thymidine Kinase and 3 C protease enzymes, with acyclovir used as the reference standard. Thymidine kinase activity was quantified using a recombinant human TK1 His-tag ELISA kit. Briefly, 50 µL of each sample or standard was pipetted into the appropriate wells. Plates were sealed and shaken at 400 rpm for 1 h at room temperature. After incubation, 100 µL of TMB development solution was added to each well, and plates were incubated in the dark at 400 rpm for 10 min. Rather than an endpoint measurement, the kinetic generation of the blue TMB product was monitored immediately at 450 nm in a microplate reader, with absorbance recorded continuously over time^43^.

3 C protease activity was assessed using a fluorogenic assay kit. The assay buffer was enriched with 0.5 M DTT to reach a final concentration of 1 mM. The frozen enzyme was thawed on ice, briefly centrifuged to collect condensation, and divided into single-use aliquots; remaining portions were stored at − 80 °C. Each well Positive Control, Inhibitor Control, and Test Sample was loaded with 30 µL of diluted protease. Blank wells received 30 µL of DTT-supplemented buffer without enzyme^44^. Then, 10 µL of inhibitor solution was added to Test Sample wells, while Positive Control wells remained inhibitor-free. The reaction was initiated by adding 10 µL of fluorogenic substrate to all wells. Plates were incubated overnight at room temperature. Fluorescence was measured at 360 nm excitation and 460 nm emission. For kinetic studies, fluorescence can be continuously monitored to track enzymatic activity over time^45^.

### Statistical analysis

All experiments were performed in triplicate, and data are presented as mean ± standard deviation (SD). Statistical analysis was conducted using one-way ANOVA, followed by Tukey’s post-hoc test for multiple comparisons. Differences were considered statistically significant at *p* < 0.05.

## Results and discussion

### Preparation and characterization of green synthesized KS-AgNPs

The green synthesis of silver nanoparticles has attracted considerable attention due to its advantages, such as the use of natural resources, rapid production, eco-friendliness, and biocompatibility. These features make them highly valuable in the pharmaceutical sector^46^. The rich array of bioactive compounds in *K. senegalensis* extract functions as both reducing and stabilizing agents, enabling the formation of various metal and metal oxide nanoparticles through favorable chemical interactions with metal precursors^32^.

The formation of KS-AgNPs was visually confirmed by a distinct color change from a clear, colorless solution to a yellowish-brown hue, indicating successful nanoparticle synthesis through surface plasmon resonance (Fig. S1).

Particle sizing (PS) is a crucial characterization method used to verify the formation of the nano-sized particles. Dynamic Light Scattering (DLS) provides the particle size in terms of hydrodynamic diameter, specifically the intensity-weighted mean diameter, also known as the z-average diameter^47^.

Furthermore, the polydispersity index (PDI) serve as a measure of the breadth of the particle size distribution. Typically, a PDI value that indicates the quality of the dispersion falls between 0 and 1^48^. KS-AgNPs showed a PDI of 0.432, indicating moderate polydispersity, which is common and acceptable, ensuring good stability and consistent antiviral activity of the synthesized nanoparticles.

Zeta potential (ZP), characterized by either a positive or negative charge depending on the particle chemistry, represents the electric potential generated by charges on the surface of particles. It serves as a measure of the repulsive forces between particles with similar charges within a formulation. These repulsive forces play a crucial role in inhibiting particle aggregation during storage. Consequently, zeta potential is a key indicator of the potential physical stability of a nano-formulation^49^.

**Fig. 1:**
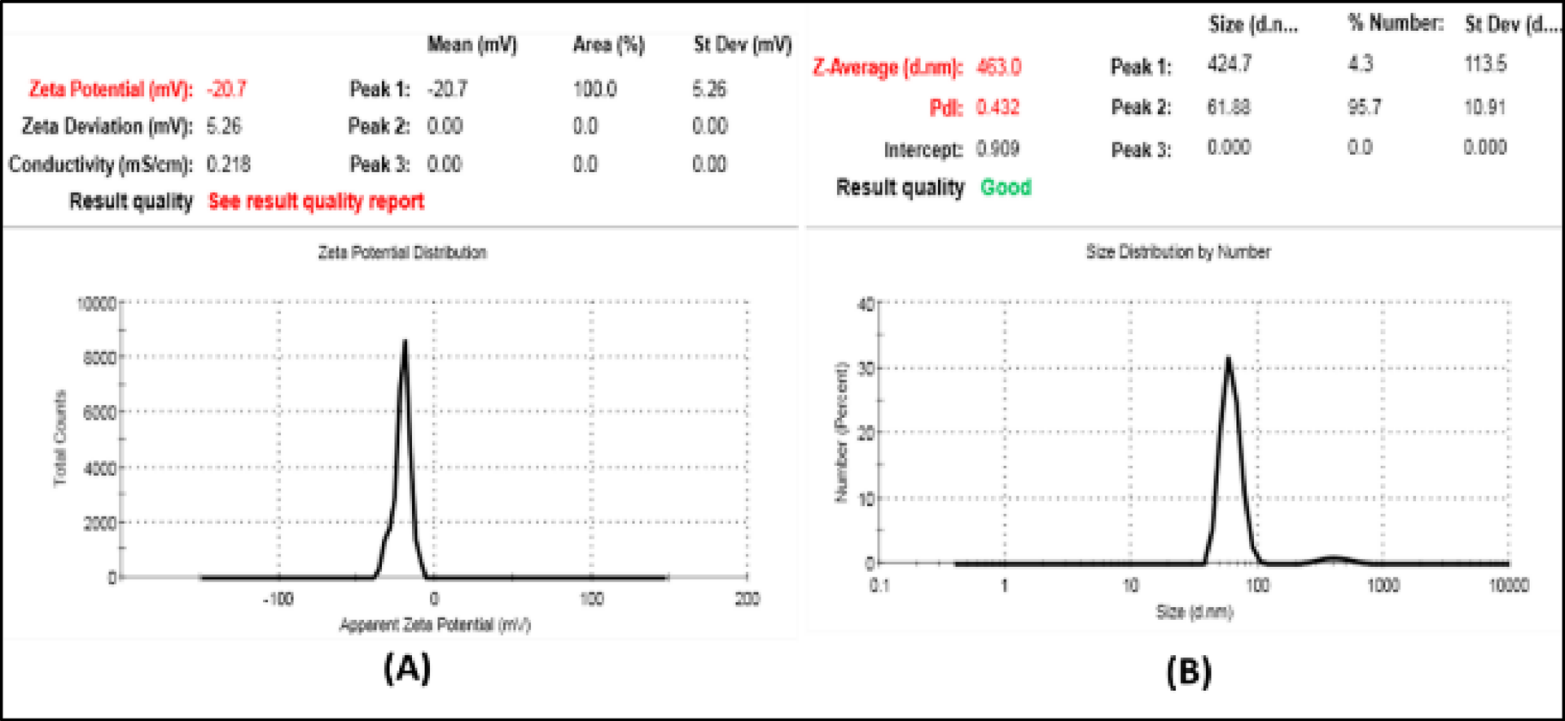
(**A**) Zeta potential analysis showing electrophoretic mobility, and (**B**) DLS profile indicating hydrodynamic size distribution of green-synthesized KS-AgNPs.

Figure 1. illustrates that the synthesized KS-AgNPs exhibited a satisfactory particle size, with a Z-average of 463 nm, a PDI of 0.432, and a ZP of (-20.7 ± 5.26). These values indicate that the prepared KS-AgNPs is homogeneous and moderate stability enhanced by steric effects from phytochemicals in the *K. senegalensis* extract.

The higher DLS Z-average (~ 463 nm) compared to the HRTEM core size (8–38 nm) represents the hydrodynamic diameter, including biomolecular coatings and solvation layers formed by adsorbed *K. senegalensis* phytochemicals. This effect, widely reported for green-synthesized AgNPs, is attributed to biomolecular corona formation and mild agglomeration rather than irreversible core aggregation^50^.

### HRTEM characterization of the green synthesis KS-AgNPs

**Fig. 2:**
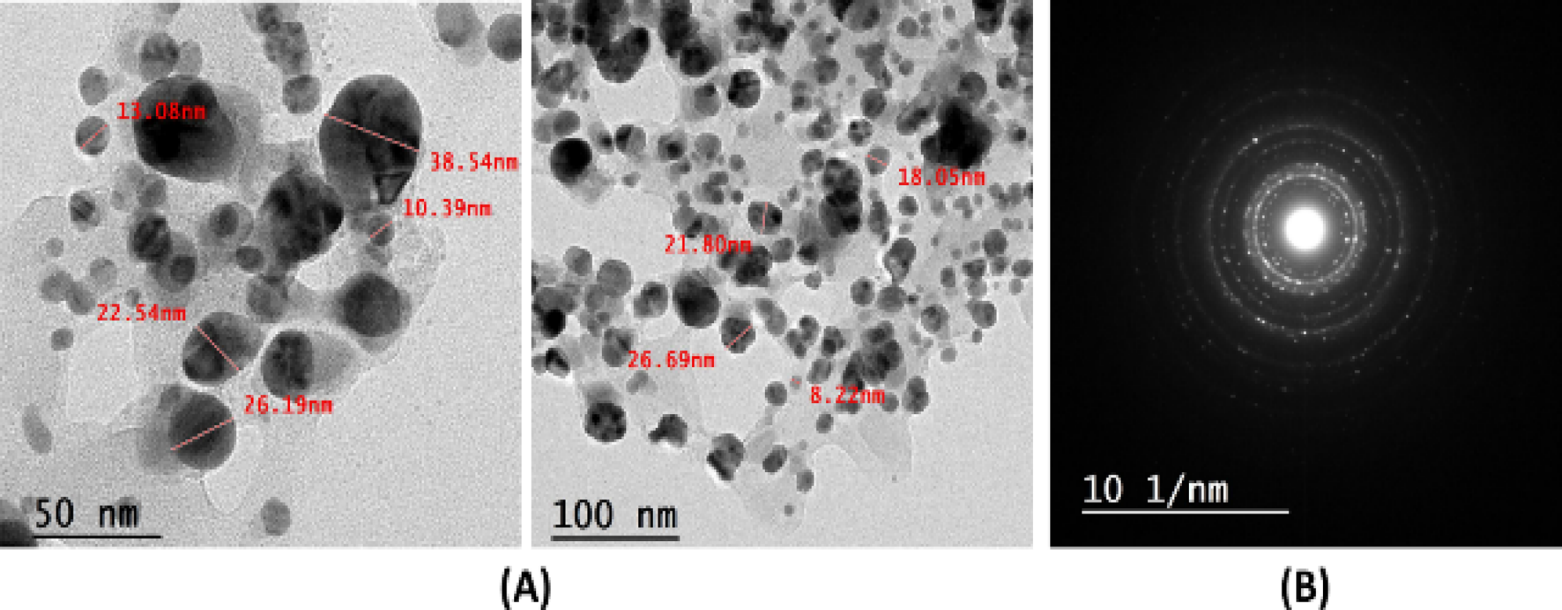
(**A**) HRTEM micrograph of KS-AgNPs illustrating morphology and size distribution. (**B**) SAED pattern confirming nanoparticles crystallinity.

The morphology of the KS-AgNPs, including their shape and size, was assessed using HRTEM analysis, as illustrated in Fig. 2A. The micrographs revealed predominantly spherical nanoparticles with diameters ranging from 8 to 38 nm. The particles were well-dispersed, no signs of agglomeration, which suggests effective stabilization by phytochemicals present in the *Khaya senegalensis* extract used during synthesis. The larger size observed by DLS (~ 463 nm) compared to TEM (8–38 nm) reflects the hydrodynamic diameter, including the core, capping biomolecules, and solvation layer, consistent with the colloidal stability shown by Zeta-sizer analysis. Furthermore, selected area electron diffraction (SAED) patterns (Fig. 2B) confirmed the crystalline nature of the synthesized Ag nanoparticles.

### UV-visible characterization of the green synthesized KS-AgNPs

**Fig. 3:**
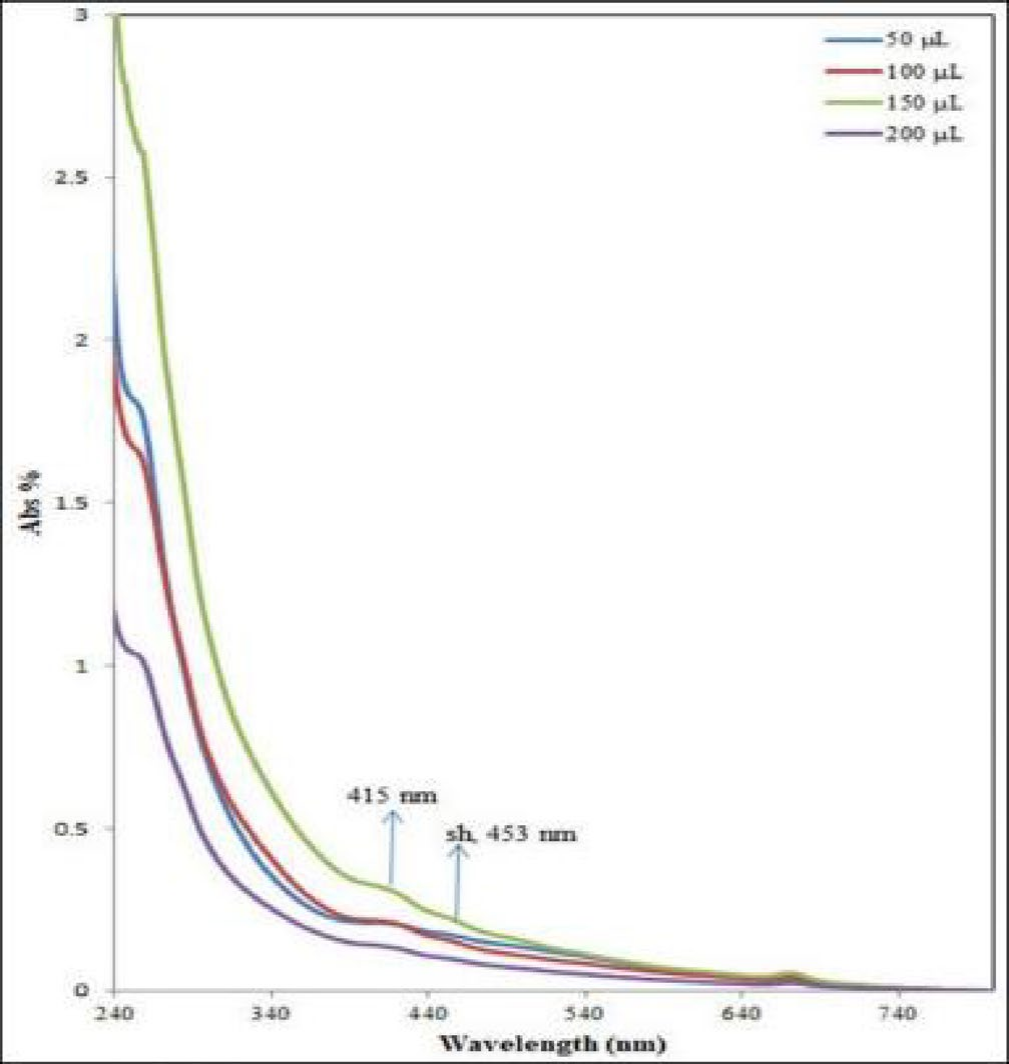
UV–vis spectra showing SPR bands of Ag nanoparticles formed at different concentrations of KS extract.

UV–Vis absorption spectrophotometry was used to monitor and confirm the formation of the green-synthesized AgNPs^51^. Silver nanoparticle suspensions are renowned for their vivid hues. In this study, the yellowish-brown coloration of the AgNPs stems from surface plasmon resonance (SPR) within the reaction mixture, providing unmistakable proof of nanoparticle formation^52^. Silver nanoparticles were synthesized by adding varying concentrations of KS methanol extract to 10 mL of a 2.5 mM AgNO₃ solution, as shown in Fig. 3. The UV–vis spectra reveal distinct absorption bands between 400 and 460 nm, corresponding to surface plasmon resonance (SPR). A rise in KS extract concentration results in increased SPR intensity, indicating more effective Ag⁺ ion reduction and greater nanoparticle yield. This is attributed to the greater availability of functional groups in the extract that facilitate both the reduction and stabilization of the nanoparticles. Additionally, maximum absorption peaks at 415 and 453 nm were observed at a concentration of 150 µL. This suggests that higher concentrations of KS extract accelerate the reaction rate, leading to a more rapid reduction of Ag^+^ ions and subsequently enhancing the nucleation rate.

### FTIR characterization of the green synthesis KS-AgNPs

**Fig. 4:**
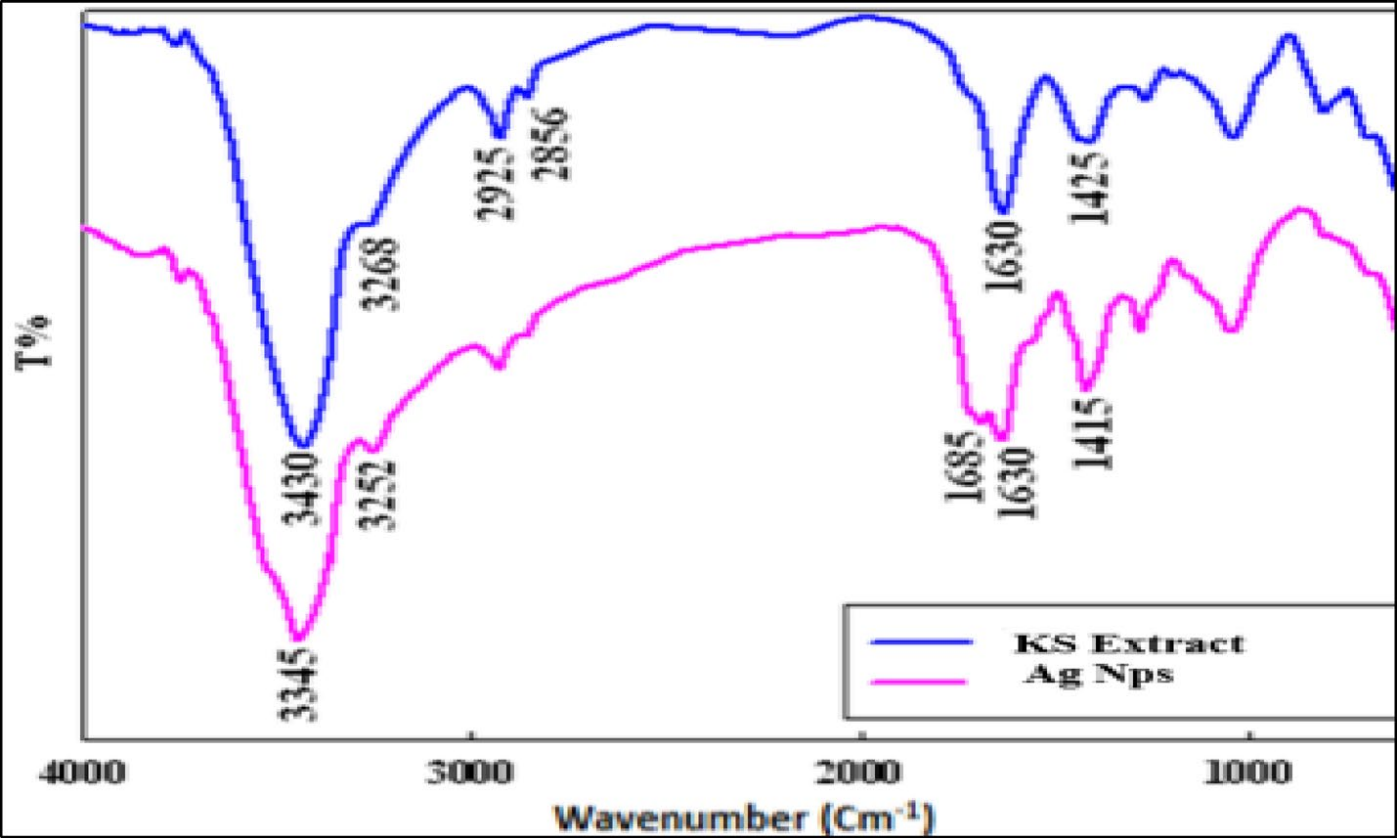
FTIR spectra of KS extract compared with that of green synthesized KS-AgNPs.

KS leaves extract contains various phytoconstituents, such as flavonoids, coumarins, sterols, polyphenols, carbohydrates, tannins, and proteins^13^. Consequently, (FTIR) serves as an effective method for identifying the functional groups involved in the reduction of silver nanoparticles. Figure 4. illustrates the FTIR spectra of the silver nanoparticles stabilized by KS extract in comparison to the original methanolic extract.

The FTIR spectrum of the K. senegalensis extract exhibits a broad band at 3430 cm⁻¹ corresponding to –OH stretching vibrations, and a signal at 3268 cm⁻¹ attributed to –NH groups from amino-containing biomolecules^53^. These functional groups are characteristic of the polyphenolic metabolites identified by UPLC-T-TOF–MS/MS, including flavonoids (e.g., myricetin, quercetin derivatives, catechin, rutin), phenolic acids (e.g., caffeic and rosmarinic acids), and coumarins (e.g., scopoletin and daphnetin), which are rich in hydroxyl and carbonyl functionalities. The bands at 2856 and 2925 cm⁻¹ correspond to aliphatic C–H stretching, while peaks at 1630 and 1425 cm⁻¹ are associated with N–H bending and phenolic O–H vibrations, respectively.

Upon interaction with AgNO₃, the observed shifts in –OH (to 3345 cm⁻¹), –NH (to 3252 cm⁻¹), and phenolic bands (1425 to 1415 cm⁻¹) indicate the direct involvement of these flavonoid-, phenolic-, and coumarin-derived functional groups in Ag⁺ reduction and nanoparticle stabilization, supporting their role as both reducing and capping agents during KS-AgNPs formation^54^.

### Metabolomics profiling study

The therapeutic potential of any medicinal plants is attributed to their diverse array of secondary metabolites or phytochemicals, which exhibit various pharmacological activities^55^. Metabolite profiling of the methanolic extract of. *K. senegalensis* leaves was conducted using UPLC/T-TOF–MS/MS. Table 1 shows the detected metabolites along with their retention times, observed molecular weights, ionization modes and chemical class. The total ion chromatograms (TIC) of *K. senegalensis* extract are displayed in Fig. S2.

**Fig. 5:**
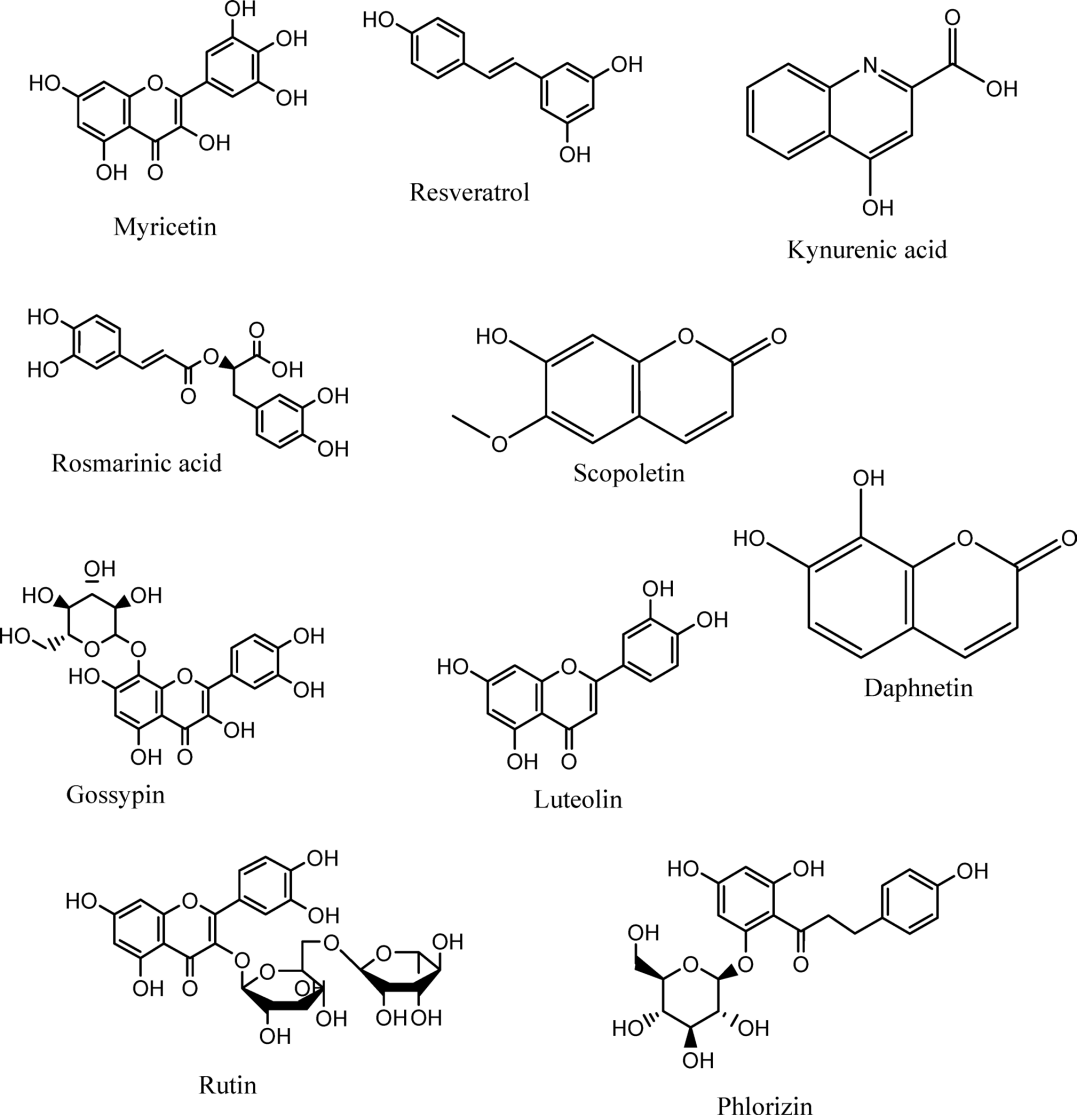
Structural illustrations of prominent metabolites such as flavonoids, phenolics, and coumarins found in *K. senegalensis* leaves extract.

Thirty metabolites were identified, with most corresponding to previously reported compounds. The extract contained flavonoid aglycones, along with O- and C-glycosides, predominantly flavones and isoflavones and their glycosylated forms, highlighting the plant’s broad biological potential^56^. Additionally, derivatives of coumarins and phenolic acids were also identified, and the structures of the major compounds are presented in Fig. 5.

**Flavonoids**

Mass ions of *m/z* 291.107, 289.089, 291.134,481.163, 611.159, 465.096, 625.171, 305.061, and 577.275 in agreement with the molecular formulas C_15_H_14_O_6_, C_15_H_12_O_6_, C_15_H_14_O_6_, C_21_H_20_O_13_, C_27_H_30_O_16_, C_21_H_20_O_12_, C_28_H_32_O_16_, C_15_H_12_O_7,_ C_30_H_26_O_12_ were identified as 3’ 4’ 5 7-pentahydroxyflavan (**5**), 3’4’5 7-tetrahydroxyflavanone (**6**), catechin (**10**), gossypin (**14**), rutin (**16**), isoquercitrin (**18**), isorhamnetin-3-O-rutinoside (**20**), Taxifolin (**21**), and Procyanidin B2 (**30**), respectively.

Apigenin 8-C-glucoside (**8**), a flavonoid-8-C-glycoside, was detected as a protonated ion [M + H]⁺ at m/z 433.1680 (C₂₁H₂₀O₁₀). quercitrin (**22**) appeared as a deprotonated ion [M − H]⁻ at m/z 447.1869. Baicalein-7-O-glucuronide (**13**), a flavonoid-7-O-glucuronide, was identified by its deprotonated ion [M − H]⁻ at m/z 445.1342, consistent with the formula C₂₁H₁₈O₁₁.

Kaempferol-3-*O*-α-L-rhamnoside (**17**) was detected at m/z 431.0996 [M-H]^−^, while luteolin-3’, 7-di-*O-*glucoside (**19**) showed a deprotonated molecule [M-H]^−^ at m/z 609.1454. Hesperetin-7-*O*-neohesperidoside (**28**) showed a deprotonated molecule [M-H]^−^ at m/z 609.1899 and phlorizin (**27**) exhibited a protonated molecule [M + H]^+^ at m/z 437.1944 with chemical formula C_21_H_24_O_10_. Phlorizin was previously reported for its significant antiviral activity against bovine viral diarrhea virus (BVDV)^57^.

Myricetin (**2**) flavonol showed a molecular ion [M-H]^−^ at m/z 317.0557. The protonated molecule [M + H] ^+^ with expected chemical formulas C_16_H_12_O_4_ and C_28_H_34_O_15_ and mass ion of *m/z* 296.0794 and 271.1681 were identified as the isoflavones formononetin (**25**) and genistein (**26**), respectively. Additionally, daidzein-8-*C*-glucoside (**24**), an isoflavone C-glycosides, appeared as a deprotonated molecule [M-H] ^−^ at mass ion of *m/z* 415.197 with chemical formula C_21_H_20_O_9_. Luteolin (**15**), a flavone, showed a molecular ion [M-H]^−^ at m/z 285.1336^58^.

Resveratrol (**4**), a stilbenoid polyphenol, was detected as a protonated ion [M + H]⁺ at m/z 229.1542.

**Phenolic acids**

The detected mass ion of *m/z* 191.0560, 179.056, 190.0471, and 291.1343 with chemical formulas of C_7_H_12_O_6_, C_9_H_8_O_4,_ C_10_H_7_NO_3,_ and C_10_H_10_O_3_ were dereplicated as quinic acid (**1**), caffeic acid (**3**), kynurenic acid (**7**), and 4-methoxy cinnamic acid (**29**), respectively. The deprotonated molecule [M-H]^−^ with expected chemical formulas C_18_H_6_O_8_ and mass ion of *m/z* 359.0975 was identified as the ester of caffeic acid, rosmarinic acid (**11**).

**Coumarins**

A tentatively identified coumarin, esculin (**9**), was detected at m/z 339.0720 of deprotonated molecule [M-H]^−^, while the observed mass ions of m/z 193.0489, and 179.1091 with chemical formulas C_10_H_8_O_4_, and C_9_H_6_O_4_ were identified as scopoletin (**12**), and daphnetin (**23**), which were previously reported in *K. senegalensis*^58^.

**Table 1:** Tentatively identified secondary metabolites identified of KS leaves methanol extract (using UPLC/T-TOF–MS/MS).

No.	Rt. (min.)	Identified metabolites	Molecular formula	Ionization mode	M/Z	MS^2^	∆ Mass (ppm)	Chemical class	References
1	10.92	Quinic acid	C_7_H_12_O_6_	Negative	191.0560	93, 85	0.3	Phenolic acids	^13^
2	11.21	Myricetin	C_15_H_10_O_8_	Negative	317.0557	271, 152, 112	– 1.2	Flavonol	^59^
3	12.02	Caffeic acid	C_9_H_8_O_4_	Negative	179.056	161,75	– 1.3	Phenolic acids	^13^
4	13.42	Resveratrol	C_14_H_12_O_3_	Positive	229.1542	142, 70	– 0.3	Stilbene	^60^
5	13.68	3’ 4’ 5 7-Pentahydroxyflavan	C_15_H_14_O_6_	Positive	291.1074	274, 159	– 0.56	Flavonoid	^13^
6	13.85	3’4’5 7-tetrahydroxyflavanone	C_15_H_12_O_6_	Positive	289.0896	203,185	– 0.4	Flavonoid	^61^
7	14.06	Kynurenic acid	C_10_H_7_NO_3_	Positive	190.0471	144, 116	0.3	Phenolic acids	^62^
8	14.53	Apigenin 8-C-glucoside	C_21_H_20_O_10_	Positive	433.1680	275	0.12	Flavonoid-8-C-glycosides	^13^
9	14.78	Esculin	C_15_H_16_O_9_	Negative	339.072	177,133	– 0.7	Coumarins glycosides	
10	15.17	Catechin	C_15_H_14_O_6_	Positive	291.1343	205,87	0.5	Flavonoid	^5^
11	15.52	Rosmarinic acid	C_18_H_6_O_8_	Negative	359.0975	239,197	0.8	Coumaric acids	^13^
12	16.23	Scopoletin	C_10_H_8_O_4_	Positive	193.0489	176,148	0.7	Coumarins glycosides	^63^
13	16.83	Baicalein-7-O-glucuronide	C_21_H_18_O_11_	Negative	445.1342	265, 239,197	– 0.4	Flavonoid-7-O-glucuronides	^13^
14	17.34	Gossypin	C_21_H_20_O_13_	Positive	481.1633	319, 147	0.13	Flavonoid	^64^
15	18.97	Luteolin	C_15_H_10_O_6_	Negative	285.1336	269, 151	0.9	Flavone	^37^
16	19.47	Rutin	C_27_H_30_O_16_	Positive	611.1597	465, 303	0.9	Flavonoid	^5,59^
17	19.59	Kaempferol-3-O-α-L-rhamnoside	C_21_H_20_O_10_	Negative	431.0996	285,227	– 0.2	Flavonoid-3-O-glycosides	^13^
18	19.97	Isoquercitrin	C_21_H_20_O_12_	Positive	465.0969	303,85	– 0.7	Flavonoid	^65^
19	20.02	Luteolin-3’, 7-di-O-glucoside	C_27_H_30_O_16_	Negative	609.1454	300,301	0.9	Flavonoid-7-O-glycosides	^13^
20	20.56	Isorhamnetin-3-O-rutinoside	C_28_H_32_O_16_	Positive	625.1711	479, 317	0.94	Flavonoid	^66^
21	20.63	Taxifolin	C_15_H_12_O_7_	Positive	305.0619	285,125	0.28	Flavonoid	^67^
22	20.82	Quercitrin	C_21_H_20_O_11_	Negative	447.1869	301, 174	0.3	Flavonoid-3-O-glycosides	^13^
23	21.58	Daphnetin	C_9_H_6_O_4_	Positive	179.1091	163,91	– 1.1	Coumarins	^68^
24	21.71	Daidzein-8-C-glucoside	C_21_H_20_O_9_	Negative	415.197	203,179	– 0.3	Isoflavonoid C-glycosides	^13^
25	21.94	Formononetin	C_16_H_12_O_4_	Positive	296.0794	287, 149	0.2	Isoflavones	^69^
26	22.36	Genistein	C_15_H_10_O_5_	Positive	271.1681	159,142	0.3	Isoflavones	^70^
27	22.69	Phlorizin	C_21_H_24_O_10_	Negative	435.1944	350,169,107	0.1	Flavonoid -O-glycosides	^71^
28	23.31	Hesperetin-7-O-neohesperidoside	C_28_H_34_O_15_	Negative	609.1899	541,499	1.3	Flavonoid-7-O-glycosides	^72^
29	23.72	4-Methoxy cinnamic acid	C_10_H_10_O_3_	Positive	179.1436	91,67	– 1.5	Phenolic acids	^73^
30	24.09	Procyanidin B2	C_30_H_26_O_12_	Positive	579.1855	561,426,409	0.3	Flavonoid	^74^

### Antiviral activity

The in vitro antiviral activity of *K. senegalensis* methanolic extract and KS-AgNPs against HSV-1 and CoxB4 was evaluated using an MTT assay. Prior to testing, cytotoxicity on Vero cells was assessed to determine safe doses. The maximum non-toxic concentrations (MNTCs) were 62.5 µg/mL for the methanol extract, 52.5 µg/mL for KS-AgNPs, and 31.2 µg/mL for the reference drug acyclovir.

**Fig. 6:**
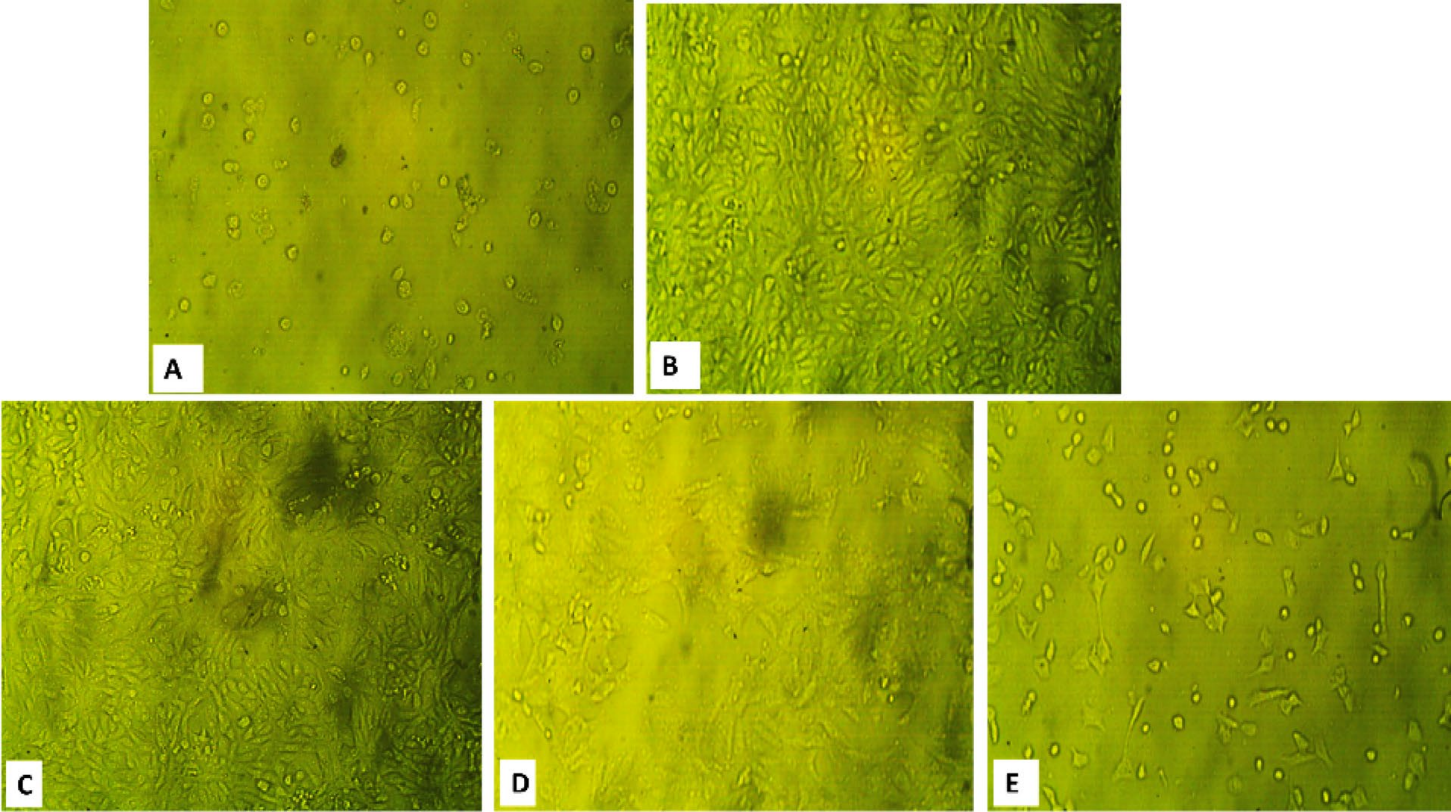
Microscopic images that illustrate the effects of (**A**) Control Vero cell; (**B**) HSV1 on Vero cell; (**C**) KS extract; (**D**) KS-AgNPs; (**E**) Acyclovir on replication of HSV-1 virus.

**Fig. 7:**
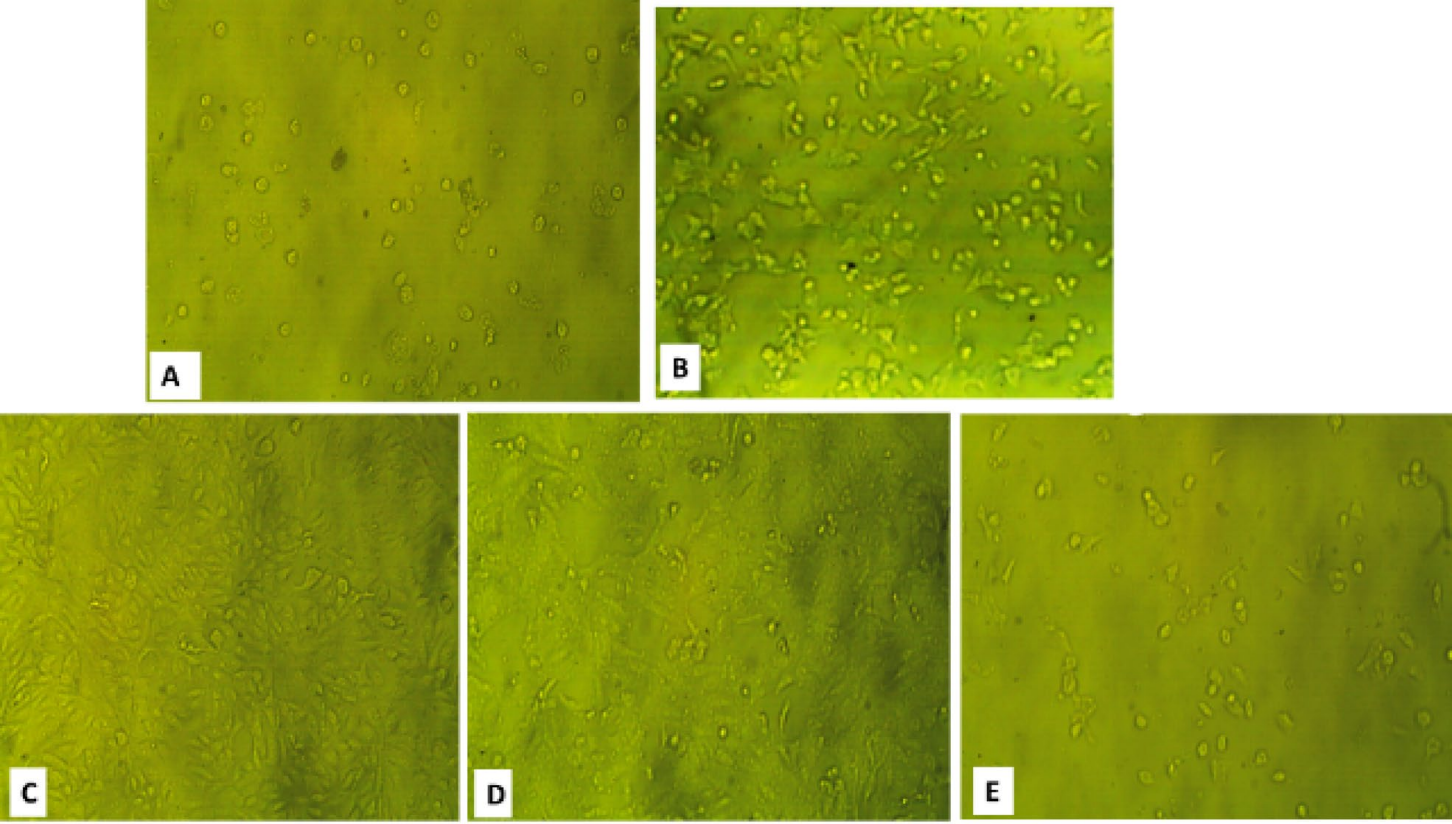
Microscopic images that illustrate the effects of (**A**) Control Vero cell; (**B**) CoxB4 on Vero cell; (**C**) KS extract; (**D**) KS-AgNPs. (**E**) Acyclovir on replication of CoxB4 virus.

The KS-AgNPs were more effective than the crude methanol extract. As shown in Tables 2 and 3; Figs. 6 and 7, the nanoparticles exhibited an IC₅₀ of 99.65 ± 1.84 µg/mL against HSV-1 and CoxB4, compared to 116.26 ± 1.28 µg/mL for the methanolic extract.

The enhanced antiviral activity of green‑synthesized silver nanoparticles (AgNPs) was likely due to the synergistic action of phytochemicals in *K. senegalensis* (e.g., flavonoids, polyphenols, coumarins) with AgNPs enhances antiviral activity by stabilizing the nanoparticles, increasing cellular uptake, and promoting multivalent interactions with viral surface proteins, which may block virus attachment and improve efficacy [75].

Al-Radadi et al.. proposed that green-synthesized AgNPs may fight viruses by producing ROS that trigger apoptosis, while bioactive molecules from plant extracts help stabilize the nanoparticles [76]. Beyond ROS generation, AgNPs can bind viral surface proteins to block attachment and entry, while myricetin interferes with HSV glycoproteins and host signaling pathways, inhibiting viral adsorption and replication [77,78].

**Table 2:** Cytotoxicity and MNTC (µg/ml) on Vero cell of KS methanol extract.

Samples	MNTC (µg/ml)	Cytotoxicity on Vero cell (µg/ml)
KS Extract	62.5	116.26 ± 1.28
KS-AgNPs	52.5	99.65 ± 1.84
Acyclovir	31.2	79.25 ± 0.14

MNTC: Maximum Non-Toxic Concentration.

**Table 3:** Antiviral effect (%) of MNTC (µg/ml) of KS methanol extract and KS-AgNPs against HSV-1 and CoxB4 viruses.

Samples	HSV-1 virus	CoxB4 virus
KS Extract	68.16	58.15
KS-AgNPs	75.73	63.38
Acyclovir	91.75	88.45

### In silico studies

#### Molecular docking

Molecular docking analysis was employed to assess the binding interactions between selected natural compounds and key viral target proteins, using both the co-crystallized ligand and the standard antiviral drug Acyclovir as reference controls. The objective was to evaluate antiviral potential based on binding affinity and overlap with benchmark interactions.

To elucidate the mechanisms underlying the antiviral activity of *K. senegalensis* extract and its silver nanoparticles (KS-AgNPs), docking studies were conducted against two clinically relevant viral enzymes: HSV-1 thymidine kinase (PDB ID: 1KI2) and Coxsackie B4 3 C protease (PDB ID: 3ZZ5). Inhibition of either enzyme impairs viral replication, making them strategic targets.

##### Computational modeling of thymidine kinase interactions

Docking validation confirmed reliability (RMSD = 1.10 Å for GA22). Reference ligands GA22 and acyclovir established benchmark hydrogen bonds and hydrophobic contacts with catalytic residues (Figs.S3 & S4). Among screened compounds, myricetin showed the most promising profile, forming diverse interactions (hydrogen bonds, π-stacking, π-cation, hydrophobic contacts) and closely mirroring the binding pattern of GA22 and acyclovir. This overlap highlights Myricetin’s potential as a primary contributor to KS antiviral activity.

**Fig. 8:**
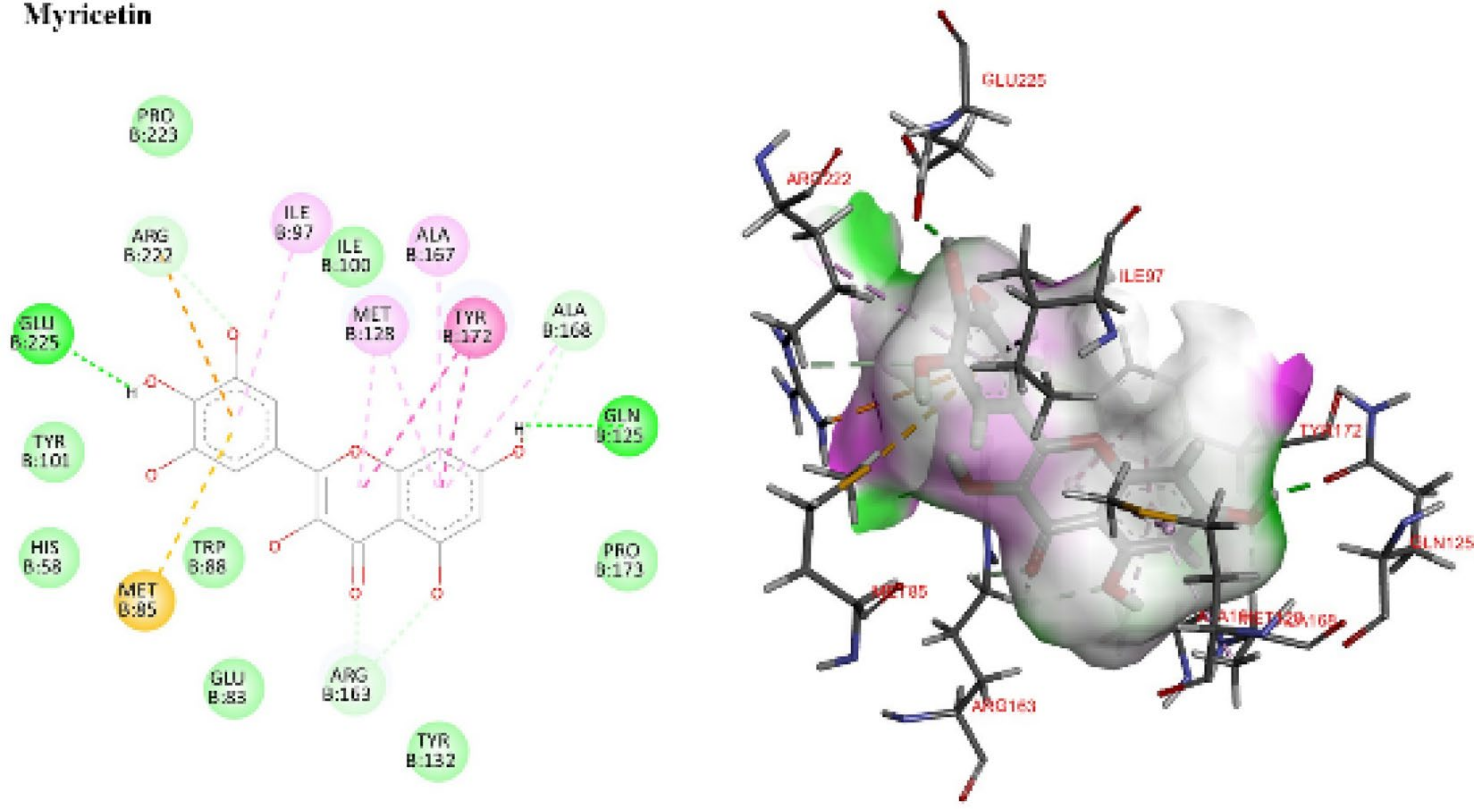
2D and 3D interaction diagrams showing key interactions between Myricetin and active site residues.

Detailed residue interactions are provided in Supplementary Table S1 and Fig. 8.

##### Computational modeling of 3 C protease

**Fig. 9:**
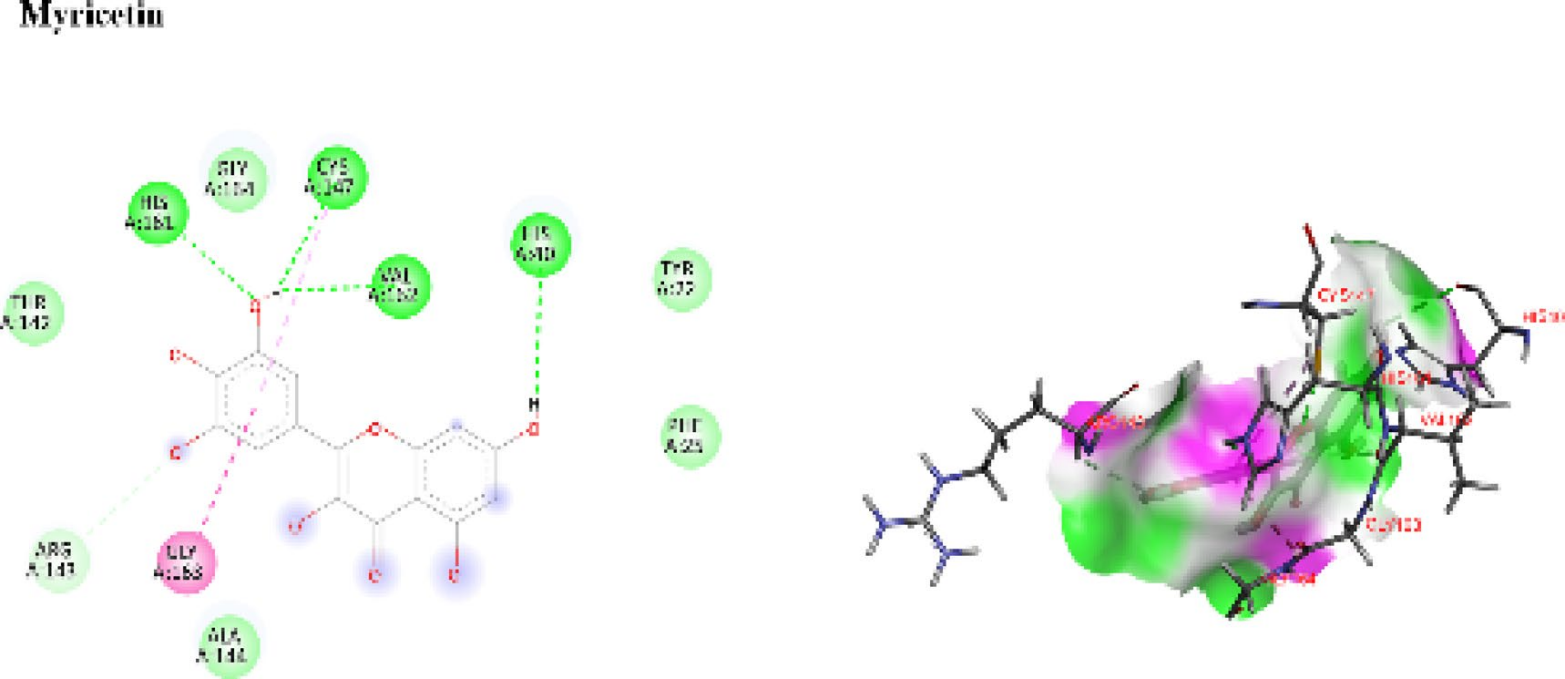
2D and 3D interaction diagrams showing key interactions between myricetin and active site residues.

Docking validation confirmed reliability (RMSD = 1.14 Å for G74). The co crystallized ligand G74 established benchmark hydrogen bonds and hydrophobic contacts within the active site (Fig. S5). Myricetin again demonstrated the most favorable binding, engaging critical catalytic residues (HIS161, CYS147, HIS40, VAL162, ARG143) and stabilizing the active site through π alkyl interactions. These interactions closely resembled those of the native ligand, indicating strong binding affinity and specificity. Detailed residue interactions are provided in Supplementary Table S2 and Fig. 9.

In conclusion, docking studies consistently identified myricetin as the lead compound, showing robust binding affinity and interaction overlap with reference ligands in both HSV-1 thymidine kinase and CoxB4 3 C protease. This supports its role as the principal contributor to the antiviral activity of *K. senegalensis*.

#### Molecular dynamics

##### Molecular dynamics analysis of thymidine kinase

Normal Mode Analysis (NMA) of HSV-1 thymidine kinase (PDB ID: 1KI2) in complex with myricetin was performed using the iMODS server, an online platform for protein flexibility and dynamics analysis. It should be noted that NMA is not a full molecular dynamics simulation but a simplified method that approximates collective motions based on harmonic models. The results revealed reduced atomic fluctuations, limited deformability near the active site, and a low eigenvalue (3.83 × 10⁻⁴), all indicative of enhanced structural stability. These findings suggest that myricetin binding reinforces conformational integrity and stabilizes functionally important regions of the enzyme. Detailed plots and variance analyses are provided in Supplementary Fig. S6.

**Molecular dynamics analysis of 3 C protease**

NMA of CoxB4 3 C protease (PDB ID: 3ZZ5) bound to myricetin was also conducted using the iMODS server. This simplified dynamics approach was selected over classical MD due to its efficiency in capturing essential flexibility patterns without requiring extensive computational resources. The analysis showed decreased flexibility compared to the crystal structure, limited motion near the active site, and a moderately low eigenvalue (9.31 × 10⁻⁴). These results indicate balanced rigidity and energy-efficient dynamics, consistent with strong ligand stabilization. Supplementary Fig. 10 presents full B-factor, deformability, covariance, and elastic network plots.

**Fig. 10:**
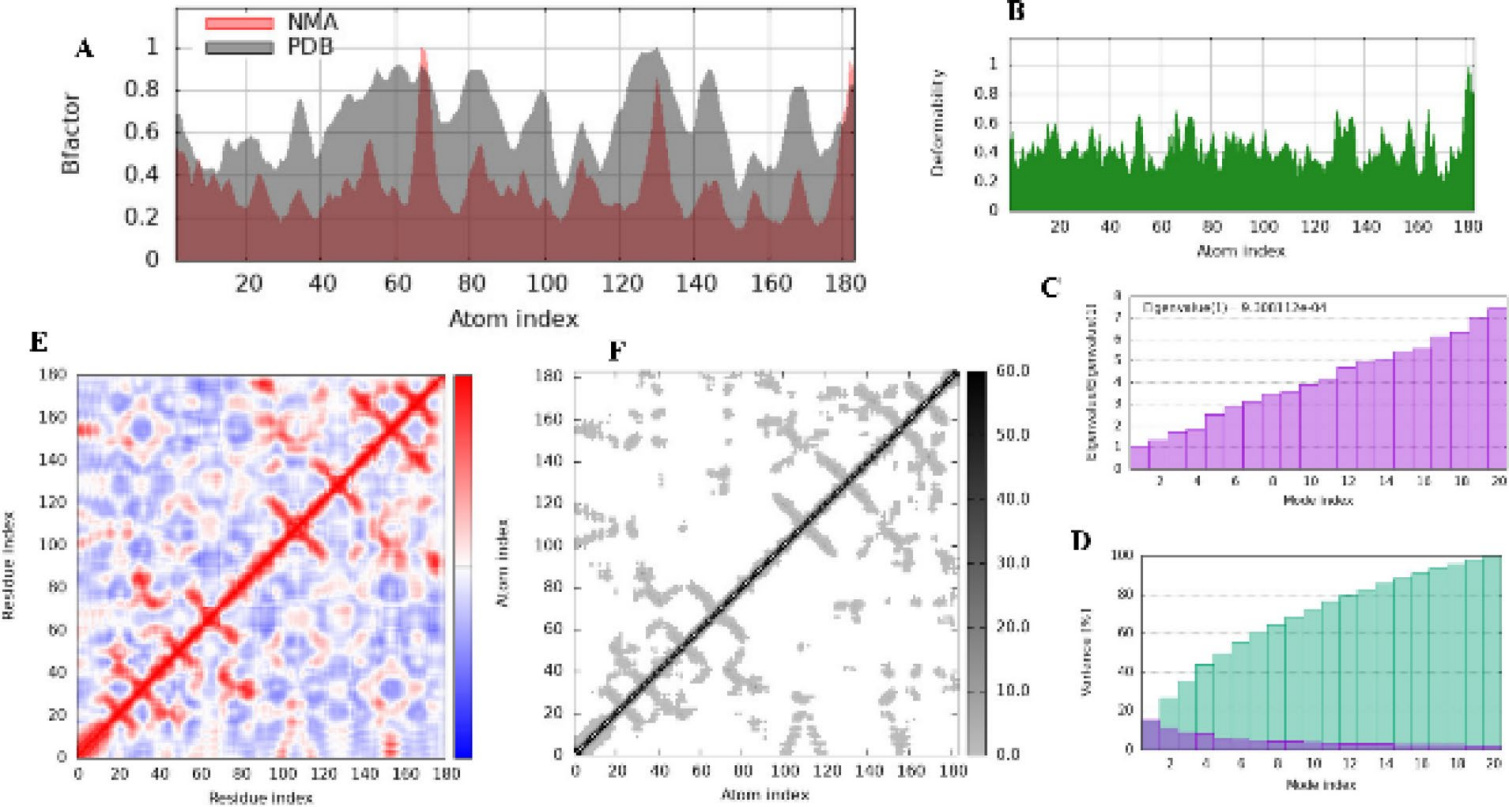
Normal Mode Analysis (NMA) results of the 3 C protease from coxsackie virus B4 (PDB ID: 3ZZ5) in complex with myricetin using the iMODS server. (**A**) Comparison of B-factor values reveals reduced flexibility post-ligand binding. (**B**) Deformability plot indicates limited motion near the active site. (**C**) Eigenvalue analysis reflects balanced rigidity and energy-efficient dynamics. (**D**) Variance profile highlights dominant low-frequency modes. (**E**) Covariance matrix illustrates both correlated and anti-correlated motions, suggesting structural communication. (**F**) Elastic network model displays high stiffness in core regions, confirming structural integrity.

In summary, Normal Mode Analyses (NMA), used here as a computationally efficient alternative to full MD simulations, confirmed that myricetin stabilizes both HSV-1 thymidine kinase and CoxB4 3 C protease, reducing flexibility while preserving essential dynamic features. This supports its potential as a promising antiviral candidate (Fig. 10).

#### Pharmacokinetics and ADME studies

To complement docking and dynamics analyses, an in silico ADME study was conducted because binding affinity alone does not guarantee therapeutic potential. Evaluating absorption, distribution, metabolism, and excretion provides early insight into drug-likeness, bioavailability, and translational feasibility. This step ensures that promising candidates such as myricetin are assessed not only for enzyme inhibition but also for their suitability as orally active antiviral agents.

In this study, the most promising compound identified through in silico molecular docking—myricetin—was selected for detailed analysis of its physicochemical properties using SwissADME. As summarized in Table S3, myricetin has a favorable molecular weight (318.24 Da) and a rigid, planar structure with minimal flexibility (1 rotatable bond, 0 sp³ carbons). It possesses strong polarity (TPSA: 151.59 Å²; 6 hydrogen bond donors), which enhances aqueous solubility but limits membrane permeability and gastrointestinal absorption.

Lipophilicity values across five predictive models indicate mild hydrophobicity, with a consensus LogP of 0.79 suggesting acceptable solubility and partial membrane permeability. However, myricetin shows low GI absorption, is not BBB permeant, and is not a P-glycoprotein substrate, while acting as an inhibitor of CYP1A2 and CYP3A4, pointing to potential metabolic interaction risks.

The predicted low GI absorption highlights a key limitation for oral delivery. Poor absorption reduces systemic exposure and therapeutic efficacy despite strong enzyme binding affinity. To address this, several strategies may be considered to improve bioavailability: (i) formulation approaches such as lipid-based carriers, nanoparticles, or cyclodextrin complexes to enhance solubility and intestinal uptake; (ii) chemical modification, including prodrug design or analogues with reduced TPSA, to improve permeability; and (iii) biological enhancers such as co-administration with permeation modifiers, enzyme inhibitors, or enteric coatings to reduce first-pass metabolism and improve systemic exposure. These approaches could help overcome polarity-driven absorption barriers.

Drug-likeness filters yield mixed results. Myricetin passes Lipinski and Ghose rules but violates Veber, Egan, and Muegge criteria due to its elevated TPSA and donor count. These properties align with its moderate bioavailability score of 0.55, indicating room for improvement through structural or formulation optimization.

Complementing this analysis, Supplementary Fig. S7 illustrates six key molecular descriptors (lipophilicity, size, polarity, insolubility, unsaturation, and flexibility) through a radar chart. Myricetin displays a balanced profile: adequate lipophilicity, manageable size, and moderate unsaturation, contributing to rigidity and π-π stacking potential. Its limited flexibility preserves binding orientation, while low insolubility supports favorable formulation characteristics.

Overall, the ADME study confirmed that; myricetin demonstrates strong structural stability and enzyme inhibitory potential, but absorption limitations highlight the need formulation strategies or structural optimization. Therefore, myricetin should be considered as a lead compound for further antiviral development rather than a ready-to-use drug candidate.

### In-vitro inhibition assay of viral thymidine kinase and 3 C protease enzymes

**Fig. 11:**
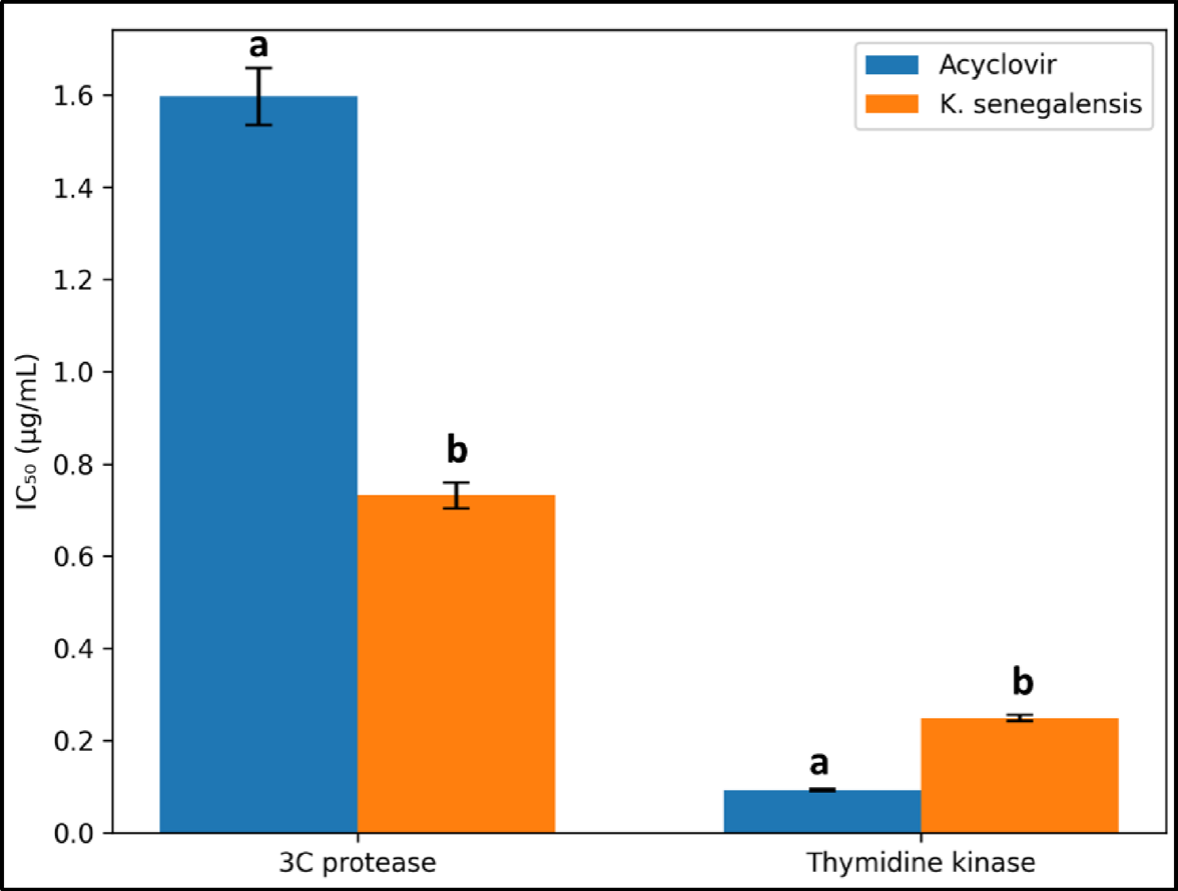
Inhibition activity (IC_50_) of *Khaya senegalensis* extract against 3 C protease and Thymidine Kinase enzymes expressed as the mean ± SD (*n* = 3). Different letters (a and b) represent statistically significant differences (*p* < 0.05) between extract and control.

Thymidine Kinase phosphorylates thymidine into dTMP, a key precursor for HSV-1 DNA replication. Its essential role in viral proliferation makes it a prime target for antiviral therapies [79]. 3 C protease (Mpro) cleaves viral polyproteins vital for Coxsackie B4 replication. Inhibitors can block this process, making them strong antiviral drug candidates [80]. Acyclovir inhibited 3 C protease and Thymidine Kinase with IC₅₀ values of 1.597 ± 0.062 and 0.092 ± 0.003 µg/mL, respectively. *K. senegalensis* methanolic extract showed stronger inhibition of 3 C protease (0.732 ± 0.028 µg/mL) and moderate inhibition of Thymidine Kinase (0.249 ± 0.007 µg/mL), as shown in Fig. 11.

## Conclusion

This study demonstrates that *Khaya senegalensis* provides a sustainable platform for the green synthesis of silver nanoparticles (KS-AgNPs). The synthesized nanoparticles exhibited antiviral activity, which is likely enhanced by the capping phytochemicals, including myricetin, that serve as reducing and stabilizing agents. Integrated computational and biological analyses suggest that both the AgNPs and the phytochemicals contribute, individually and synergistically, to viral enzyme inhibition. These findings highlight AgNPs loaded *Khaya senegalensis* as promising candidates for antiviral nano-therapeutics, providing mechanistic insights and as a basis for future studies against drug-resistant viral strains.

## Future perspectives and limitations of the study

Although the present study reported promising antiviral and enzymatic inhibition activities, certain limitations should be noted. Future studies would include expanded antiviral screening (e.g. SARS-COV-2, H1N1, HIV, and hepatitis viruses) and address scalability, stability, and in vivo safety to facilitate translational development. In addition, virological parameters such as the selectivity index (SI) were not assessed, and X-ray diffraction (XRD) analysis was confined to nanoparticle phase identification. Moreover, comparative metabolomic profiling of the crude extract and synthesized KS-AgNPs was not conducted.

## Supplementary Information

Below is the link to the electronic supplementary material.

Supplementary Material 1

## Data Availability

All data are contained within this article and the Supplementary Material.
