# Supplementary material for: Green silver nanoparticles of Khaya senegalensis as dual inhibitors of viral thymidine kinase and 3 C protease: metabolomics, and computational insights
Source: Sci Rep. 2026 Mar 29;16:10527. doi: 10.1038/s41598-026-43691-6 (PMC13036088; doi:10.1038/s41598-026-43691-6)
Supplement: Supplementary file 1 — Supplementary Material 1 [file 41598_2026_43691_MOESM1_ESM.docx]

**Supplementary Materials**

**Green Silver Nanoparticles of *Khaya senegalensis* as Dual Inhibitors of Viral thymidine kinase and 3C protease: Metabolomics, and computational insights**

Heba A. El Gizawy^1*^, Rehab H. Abd El-Aleam ^2^ and Nevine H. Hassan^3*^

^1^ Department of Pharmacognosy, Faculty of Pharmacy, October 6 University, 6^th^ of October City, Giza, 12585, Egypt

^2^ Pharmaceutical Chemistry Department, Faculty of Pharmacy, Modern University for Technology and Information MTI, Cairo 11571, Egypt

^3^ Department of Pharmacognosy, Faculty of Pharmacy, Modern University for Technology and Information, Cairo 11571, Egypt

*Corresponding authors: Heba A. El Gizawy ([hebaelgizawy@o6u.edu.eg](mailto:hebaelgizawy@o6u.edu.eg)) , and Nevine H. Hassan ([Nevine.Hossam@pharm.mti.edu.eg](mailto:Nevine.Hossam@pharm.mti.edu.eg))


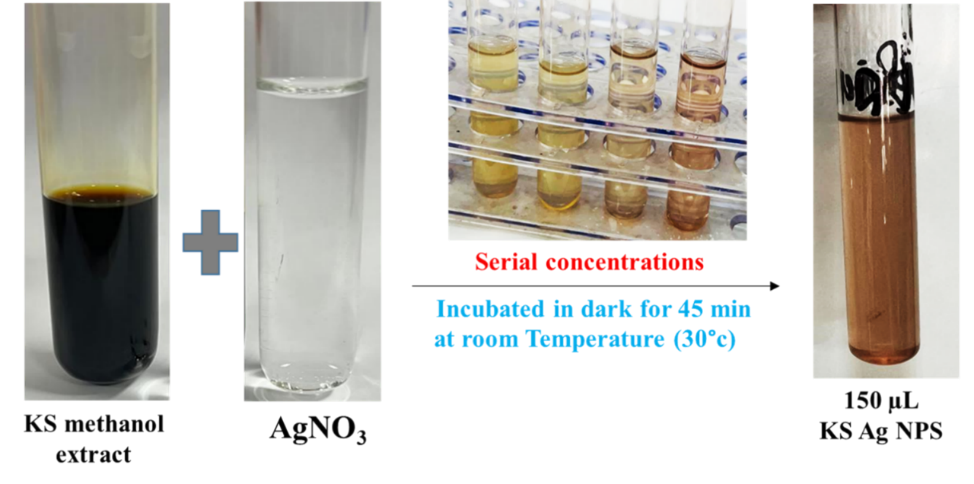


**Figure S1.** Formation of AgNPs in KS methanol extract observed as color change to yellowish brown after 45 min. incubation with 2.5 mM AgNO_3_ solution

**
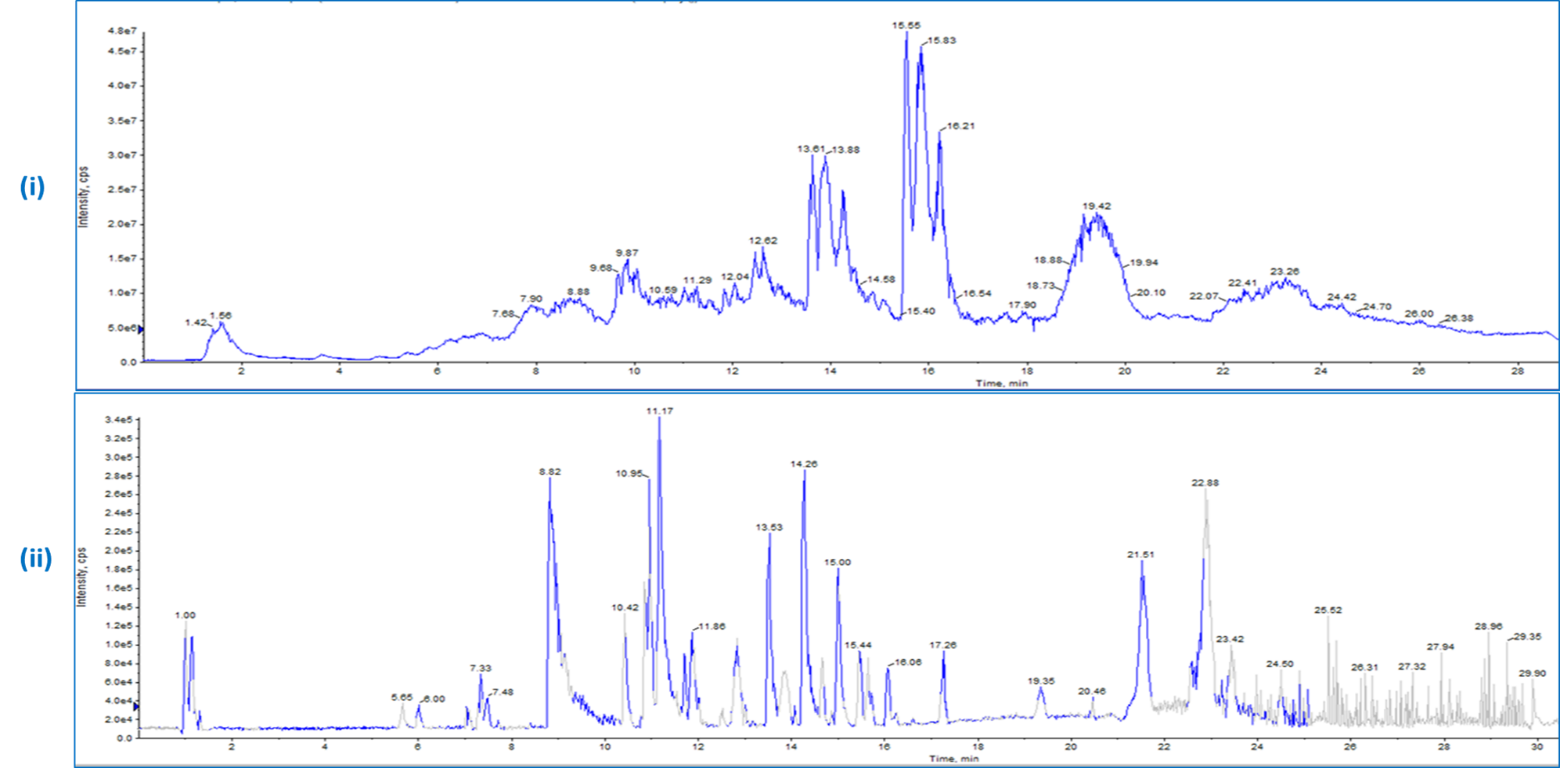
**

**Figure S2.** Total ion chromatogram of KS methanol extract recorded in (i) positive ionization mode and (ii) negative ionization mode.


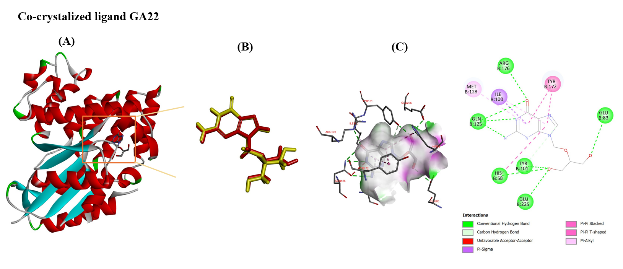


**Figure S3.** Validation of docking protocol and interaction analysis of GA22 with thymidine kinase. **(A)** Overall 3D structure of thymidine kinase complexed with GA22**. (B)** Superimposition of the docked pose with the native co-crystallized GA22 (RMSD = 1.10 Å), confirming docking accuracy**. (C)** 3D and 2D interaction diagrams showing key interactions between GA22 and active site residues.


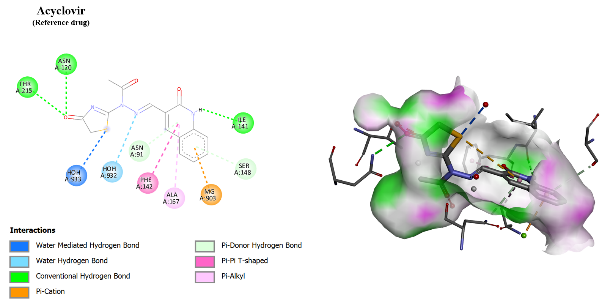


**Figure S4:** 2D and 3D interaction diagrams showing key interactions between acyclovir and active site residues.


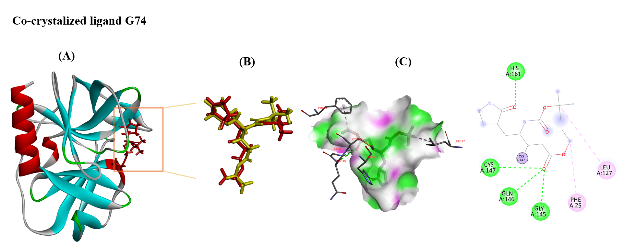


**Figure S5.** Validation of docking protocol and interaction analysis of G74 with 3C protease CoxB4 virus. **(A)** Overall 3D structure of 3C protease complexed with G74. **(B)** Superimposition of the docked pose with the native co-crystallized GA22 (RMSD = 1.14 Å), confirming docking accuracy. **(C)** 3D and 2D interaction diagrams showing key interactions between G74 and active site residues.

**
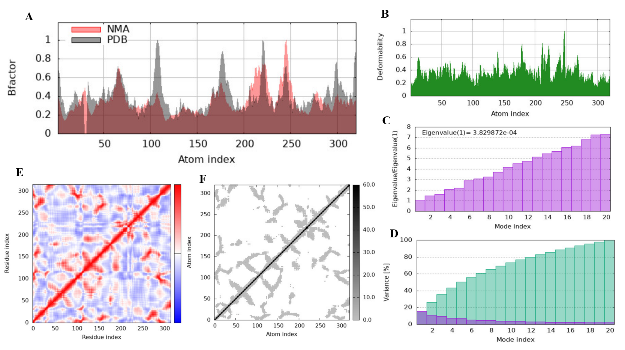
**

**Figure S6:** Normal Mode Analysis (NMA) of thymidine kinase from Herpes Simplex Virus type I (PDB ID: 1KI2) in complex with myricetin, illustrating dynamic and structural stability parameters obtained via the iMODS server. **(A)** B-factor comparison showing reduced atomic fluctuations upon ligand binding. **(B)** Deformability plot identifying flexible regions within the protein. **(C)** Eigenvalue distribution, with the first eigenvalue of 3.829872 × 10⁻⁴ indicating complex stability. **(D)** Variance contribution per mode reflecting dominant collective motions. **(E)** Covariance matrix depicting residue correlation patterns. **(F)** Elastic network model highlighting interatomic stiffness in core regions.


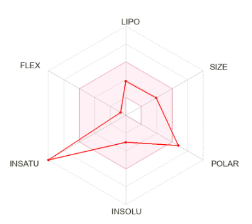


**Figure S7.** Bioavailability Radar, showing myricetin, represented by central red lines fit in the pink area, which is the optimal range for each property.

**Table S1:** Summary of Docking Energies and Interaction Profiles for Compounds identified from *Khaya senegalensis extract* Compared to acyclovir and GA22, in thymidine kinase

| **Compounds** | **Docking Score (kcal/mol)** | **Amino acid** | **Distance** | **Key Interaction** | |
| --- | --- | --- | --- | --- | --- |
|  |  |  |  | **Category** | **Types** |
| **Co-crystalized ligand (GA22)** | -10.54 | HIS58 | 2.99235 | Hydrogen Bond | Conventional Hydrogen Bond |
|  |  | TYR101 | 2.53294 | Hydrogen Bond | Conventional Hydrogen Bond |
|  |  | GLN125 | 3.03878 | Hydrogen Bond | Conventional Hydrogen Bond |
|  |  | ARG176 | 2.83199 | Hydrogen Bond | Conventional Hydrogen Bond |
|  |  | GLU83 | 3.37216 | Hydrogen Bond | Conventional Hydrogen Bond |
|  |  | ILE100 | 3.9664 | Hydrophobic | Pi-Sigma |
|  |  | TYR172 | 4.27799 | Hydrophobic | Pi-Pi Stacked |
|  |  | HIS58 | 5.68887 | Hydrophobic | Pi-Pi T-shaped |
|  |  | MET128 | 4.69995 | Hydrophobic | Pi-Alkyl |
|  |  | ILE100 | 5.09579 | Hydrophobic | Pi-Alkyl |
| **Acyclovir** | -10.71 | GLN125 | 2.20898 | Hydrogen Bond | Conventional Hydrogen Bond |
|  |  | GLU225 | 2.03239 | Hydrogen Bond | Conventional Hydrogen Bond |
|  |  | ALA168 | 3.00963 | Hydrogen Bond | Carbon Hydrogen Bond |
|  |  | GLU225 | 2.73653 | Hydrogen Bond | Carbon Hydrogen Bond |
|  |  | TYR172 | 3.93682 | Hydrophobic | Pi-Pi Stacked |
|  |  | TYR172 | 3.81664 | Hydrophobic | Pi-Pi Stacked |
|  |  | ILE100 | 5.25181 | Hydrophobic | Pi-Alkyl |
|  |  | MET128 | 4.2586 | Hydrophobic | Pi-Alkyl |
| **Myricetin** | -10.98 | GLN125 | 1.58039 | Hydrogen Bond | Conventional Hydrogen Bond |
|  |  | GLU225 | 1.6938 | Hydrogen Bond | Conventional Hydrogen Bond |
|  |  | ARG163 | 2.35673 | Hydrogen Bond | Carbon Hydrogen Bond |
|  |  | ARG163 | 2.45698 | Hydrogen Bond | Carbon Hydrogen Bond |
|  |  | ARG163 | 2.16937 | Hydrogen Bond | Carbon Hydrogen Bond |
|  |  | ALA168 | 2.33034 | Hydrogen Bond | Carbon Hydrogen Bond |
|  |  | ARG222 | 2.94686 | Hydrogen Bond | Carbon Hydrogen Bond |
|  |  | ARG222 | 4.53912 | Electrostatic | Pi-Cation |
|  |  | MET85 | 5.91908 | Other | Pi-Sulfur |
|  |  | TYR172 | 4.71128 | Hydrophobic | Pi-Pi Stacked |
|  |  | TYR172 | 4.0908 | Hydrophobic | Pi-Pi Stacked |
|  |  | MET128 | 4.5813 | Hydrophobic | Pi-Alkyl |
|  |  | ALA167 | 5.25296 | Hydrophobic | Pi-Alkyl |
|  |  | ALA168 | 4.55978 | Hydrophobic | Pi-Alkyl |
|  |  | ILE97 | 4.12968 | Hydrophobic | Pi-Alkyl |
|  |  | ARG222 | 4.9138 | Hydrophobic | Pi-Alkyl |
| **Resveratrol** | -10.3 | ARG176 | 2.25979 | Hydrogen Bond | Conventional Hydrogen Bond |
|  |  | ARG222 | 2.38628 | Hydrogen Bond | Conventional Hydrogen Bond |
|  |  | GLN125 | 1.77207 | Hydrogen Bond | Conventional Hydrogen Bond |
|  |  | ARG222 | 3.5122 | Electrostatic | Pi-Cation |
|  |  | TYR172 | 4.08034 | Hydrophobic | Pi-Pi Stacked |
|  |  | ARG222 | 5.1725 | Hydrophobic | Pi-Alkyl |
|  |  | ILE100 | 4.88377 | Hydrophobic | Pi-Alkyl |
|  |  | MET128 | 4.64379 | Hydrophobic | Pi-Alkyl |
| **Kynurenic acid** | -10.2 | HIS58 | 2.89286 | Hydrogen Bond | Conventional Hydrogen Bond |
|  |  | TYR101 | 2.75629 | Hydrogen Bond | Conventional Hydrogen Bond |
|  |  | ARG163 | 3.39409 | Hydrogen Bond | Conventional Hydrogen Bond |
|  |  | MET128 | 3.89752 | Hydrophobic | Pi-Sigma |
|  |  | TYR172 | 3.87565 | Hydrophobic | Pi-Pi Stacked |
|  |  | ALA168 | 5.28605 | Hydrophobic | Pi-Alkyl |
| **Rosmarinic acid** | -10.44 | HIS58 | 2.73427 | Hydrogen Bond | Conventional Hydrogen Bond |
|  |  | TYR101 | 2.8186 | Hydrogen Bond | Conventional Hydrogen Bond |
|  |  | GLN125 | 3.02097 | Hydrogen Bond | Conventional Hydrogen Bond |
|  |  | ARG176 | 3.34744 | Hydrogen Bond | Conventional Hydrogen Bond |
|  |  | ARG222 | 2.79021 | Hydrogen Bond | Conventional Hydrogen Bond |
|  |  | GLU83 | 1.94899 | Hydrogen Bond | Conventional Hydrogen Bond |
|  |  | ILE100 | 3.65687 | Hydrophobic | Pi-Sigma |
|  |  | MET128 | 3.3801 | Hydrophobic | Pi-Sigma |
|  |  | MET128 | 5.80373 | Other | Pi-Sulfur |
|  |  | TYR172 | 4.68046 | Hydrophobic | Pi-Pi Stacked |
|  |  | ALA168 | 4.88461 | Hydrophobic | Pi-Alkyl |
| **Scopoletin** | -9.89 | ARG176 | 3.18316 | Hydrogen Bond | Conventional Hydrogen Bond |
|  |  | GLN125 | 2.00236 | Hydrogen Bond | Conventional Hydrogen Bond |
|  |  | TYR132 | 3.61273 | Hydrogen Bond | Carbon Hydrogen Bond |
|  |  | MET128 | 3.52713 | Hydrophobic | Pi-Sigma |
|  |  | TYR172 | 3.96271 | Hydrophobic | Pi-Pi Stacked |
|  |  | ALA167 | 4.31606 | Hydrophobic | Alkyl |
|  |  | ARG163 | 4.50102 | Hydrophobic | Alkyl |
|  |  | TRP88 | 4.74832 | Hydrophobic | Pi-Alkyl |
|  |  | TYR132 | 4.73365 | Hydrophobic | Pi-Alkyl |
|  |  | ILE100 | 5.42893 | Hydrophobic | Pi-Alkyl |
|  |  | MET128 | 5.43504 | Hydrophobic | Pi-Alkyl |
| **Gossypin** | -10.1 | ARG176 | 2.89055 | Hydrogen Bond | Conventional Hydrogen Bond |
|  |  | ARG222 | 2.9722 | Hydrogen Bond | Conventional Hydrogen Bond |
|  |  | GLU225 | 1.91892 | Hydrogen Bond | Conventional Hydrogen Bond |
|  |  | ARG163 | 2.08294 | Hydrogen Bond | Carbon Hydrogen Bond |
|  |  | GLN125 | 2.2874 | Hydrogen Bond | Carbon Hydrogen Bond |
|  |  | GLN125 | 2.63859 | Hydrogen Bond | Carbon Hydrogen Bond |
|  |  | TYR101 | 3.2313 | Hydrogen Bond | Pi-Donor Hydrogen Bond |
|  |  | HIS58 | 5.36076 | Hydrophobic | Pi-Pi T-shaped |
|  |  | ILE97 | 4.7505 | Hydrophobic | Pi-Alkyl |
|  |  | ARG222 | 4.90893 | Hydrophobic | Pi-Alkyl |
|  |  | MET128 | 3.8951 | Hydrophobic | Pi-Alkyl |
|  |  | ALA168 | 4.43605 | Hydrophobic | Pi-Alkyl |
| **Luteolin** | -9.93 | ARG176 | 3.14746 | Hydrogen Bond | Conventional Hydrogen Bond |
|  |  | TYR101 | 2.79476 | Hydrogen Bond | Conventional Hydrogen Bond |
|  |  | GLU225 | 1.71507 | Hydrogen Bond | Conventional Hydrogen Bond |
|  |  | GLN125 | 2.2977 | Hydrogen Bond | Conventional Hydrogen Bond |
|  |  | MET128 | 3.68836 | Hydrophobic | Pi-Sigma |
|  |  | TYR172 | 4.06338 | Hydrophobic | Pi-Pi Stacked |
|  |  | TYR172 | 3.9147 | Hydrophobic | Pi-Pi Stacked |
|  |  | HIS58 | 4.51148 | Hydrophobic | Pi-Pi T-shaped |
|  |  | TRP88 | 5.90153 | Hydrophobic | Pi-Pi T-shaped |
|  |  | ILE100 | 5.0115 | Hydrophobic | Pi-Alkyl |
|  |  | MET128 | 4.7637 | Hydrophobic | Pi-Alkyl |
|  |  | ARG222 | 4.8294 | Hydrophobic | Pi-Alkyl |
| **Rutin** | -10.13 | MET60 | 2.57729 | Hydrogen Bond | Conventional Hydrogen Bond |
|  |  | ARG163 | 3.06195 | Hydrogen Bond | Conventional Hydrogen Bond |
|  |  | ARG220 | 2.95673 | Hydrogen Bond | Conventional Hydrogen Bond |
|  |  | ARG222 | 1.80061 | Hydrogen Bond | Conventional Hydrogen Bond |
|  |  | ARG216 | 1.73624 | Hydrogen Bond | Conventional Hydrogen Bond |
|  |  | GLU225 | 2.18894 | Hydrogen Bond | Conventional Hydrogen Bond |
|  |  | HIS58 | 2.44614 | Hydrogen Bond | Carbon Hydrogen Bond |
|  |  | ALA168 | 2.77495 | Hydrogen Bond | Carbon Hydrogen Bond |
|  |  | GLU83 | 2.50284 | Hydrogen Bond | Carbon Hydrogen Bond |
|  |  | TYR172 | 2.48612 | Hydrogen Bond | Pi-Donor Hydrogen Bond |
|  |  | ALA167 | 3.67426 | Hydrophobic | Alkyl |
|  |  | ALA168 | 3.93352 | Hydrophobic | Alkyl |
|  |  | TYR172 | 4.3279 | Hydrophobic | Pi-Alkyl |
|  |  | ARG220 | 5.38224 | Hydrophobic | Pi-Alkyl |
|  |  | LEU217 | 4.11169 | Hydrophobic | Pi-Alkyl |
|  |  | ARG222 | 5.3603 | Hydrophobic | Pi-Alkyl |
| **Daphnetin** | -9.81 | HIS58 | 2.88874 | Hydrogen Bond | Conventional Hydrogen Bond |
|  |  | TYR101 | 2.95095 | Hydrogen Bond | Conventional Hydrogen Bond |
|  |  | TYR101 | 3.14519 | Hydrogen Bond | Conventional Hydrogen Bond |
|  |  | ARG176 | 3.18941 | Hydrogen Bond | Conventional Hydrogen Bond |
|  |  | ARG176 | 2.80027 | Hydrogen Bond | Conventional Hydrogen Bond |
|  |  | TYR172 | 4.03164 | Hydrophobic | Pi-Pi Stacked |
|  |  | TYR172 | 4.25554 | Hydrophobic | Pi-Pi Stacked |
|  |  | ILE100 | 4.87031 | Hydrophobic | Pi-Alkyl |
|  |  | MET128 | 5.0839 | Hydrophobic | Pi-Alkyl |
| **Phlorizin** | -10.22 | LYS62 | 2.4491 | Hydrogen Bond | Conventional Hydrogen Bond |
|  |  | THR63 | 1.98999 | Hydrogen Bond | Conventional Hydrogen Bond |
|  |  | ARG163 | 2.27486 | Hydrogen Bond | Conventional Hydrogen Bond |
|  |  | ARG176 | 2.01478 | Hydrogen Bond | Conventional Hydrogen Bond |
|  |  | GLU225 | 1.71073 | Hydrogen Bond | Conventional Hydrogen Bond |
|  |  | GLN125 | 2.40448 | Hydrogen Bond | Conventional Hydrogen Bond |
|  |  | ARG222 | 3.00981 | Electrostatic | Pi-Cation |
|  |  | HIS58 | 5.53832 | Hydrophobic | Pi-Pi Stacked |
|  |  | ILE97 | 4.64522 | Hydrophobic | Pi-Alkyl |
|  |  | ARG222 | 4.67026 | Hydrophobic | Pi-Alkyl |
|  |  | LYS62 | 4.55433 | Hydrophobic | Pi-Alkyl |

**Table S2:** Summary of Docking Energies and Interaction Profiles for Compounds identified from *Khaya senegalensis* extract Compared to **G74**, in 3C protease.

| **Compounds** | **Docking Score (kcal/mol)** | **Distance** | **Amino acid** | **Key Interactions** | |
| --- | --- | --- | --- | --- | --- |
|  |  |  |  | **Category** | **Types** |
| **G74**  **Co-Crystalized ligand** | -9.51 | 2.75437 | GLY145 | Hydrogen Bond | Conventional Hydrogen Bond |
|  |  | 2.03757 | HIS161 | Hydrogen Bond | Conventional Hydrogen Bond |
|  |  | 3.37112 | GLN146 | Hydrogen Bond | Conventional Hydrogen Bond |
|  |  | 3.31164 | CYS147 | Hydrogen Bond | Conventional Hydrogen Bond |
|  |  | 5.4411 | LEU127 | Hydrophobic | Alkyl |
|  |  | 4.45167 | PHE25 | Hydrophobic | Pi-Alkyl |
| **Myricetin** | -9.42 | 2.53958 | HIS161 | Hydrogen Bond | Conventional Hydrogen Bond |
|  |  | 3.09355 | HIS40 | Hydrogen Bond | Conventional Hydrogen Bond |
|  |  | 2.59424 | CYS147 | Hydrogen Bond | Conventional Hydrogen Bond |
|  |  | 2.76972 | VAL162 | Hydrogen Bond | Conventional Hydrogen Bond |
|  |  | 3.07174 | ARG143 | Hydrogen Bond | Carbon Hydrogen Bond |
|  |  | 5.37789 | CYS147 | Hydrophobic | Pi-Alkyl |
| **Resveratrol** | -9.10 | 2.1459 | ARG143 | Hydrogen Bond | Conventional Hydrogen Bond |
|  |  | 5.15034 | CYS147 | Other | Pi-Sulfur |
|  |  | 4.97047 | HIS40 | Hydrophobic | Pi-Pi T-shaped |
|  |  | 4.76966 | LEU127 | Hydrophobic | Pi-Alkyl |
| **Kynurenic acid** | -8.98 | 2.28529 | HIS161 | Hydrogen Bond | Conventional Hydrogen Bond |
|  |  | 2.47517 | CYS147 | Hydrogen Bond | Conventional Hydrogen Bond |
|  |  | 4.83993 | CYS147 | Other | Pi-Sulfur |
|  |  | 3.9983 | ARG143 | Hydrophobic | Amide-Pi Stacked |
|  |  | 4.17364 | ALA144 | Hydrophobic | Pi-Alkyl |
| **Rosmarinic acid** | -8.75 | 2.91943 | GLU24 | Hydrogen Bond | Conventional Hydrogen Bond |
|  |  | 3.38313 | CYS147 | Hydrogen Bond | Conventional Hydrogen Bond |
|  |  | 2.55229 | HIS161 | Hydrogen Bond | Conventional Hydrogen Bond |
|  |  | 2.10001 | VAL162 | Hydrogen Bond | Conventional Hydrogen Bond |
|  |  | 2.42998 | TYR22 | Hydrogen Bond | Conventional Hydrogen Bond |
|  |  | 3.06106 | THR142 | Hydrogen Bond | Carbon Hydrogen Bond |
|  |  | 2.6741 | GLY164 | Hydrogen Bond | Carbon Hydrogen Bond |
|  |  | 5.31249 | ALA144 | Hydrophobic | Pi-Alkyl |
| **Scopoletin** | -8.91 | 2.1637 | HIS161 | Hydrogen Bond | Conventional Hydrogen Bond |
|  |  | 2.44671 | VAL162 | Hydrogen Bond | Conventional Hydrogen Bond |
|  |  | 2.45824 | THR142 | Hydrogen Bond | Carbon Hydrogen Bond |
|  |  | 5.16604 | CYS147 | Other | Pi-Sulfur |
| **Gossypin** | -8.54 | 3.01752 | LYS42 | Hydrogen Bond | Conventional Hydrogen Bond |
|  |  | 3.35872 | CYS147 | Hydrogen Bond | Conventional Hydrogen Bond |
|  |  | 3.19567 | CYS147 | Hydrogen Bond | Conventional Hydrogen Bond |
|  |  | 2.33675 | HIS161 | Hydrogen Bond | Conventional Hydrogen Bond |
|  |  | 2.02336 | GLU24 | Hydrogen Bond | Conventional Hydrogen Bond |
|  |  | 2.54299 | TYR22 | Hydrogen Bond | Conventional Hydrogen Bond |
|  |  | 2.38442 | CYS147 | Hydrogen Bond | Conventional Hydrogen Bond |
|  |  | 2.84738 | GLY164 | Hydrogen Bond | Carbon Hydrogen Bond |
|  |  | 5.98417 | CYS147 | Other | Pi-Sulfur |
|  |  | 3.70686 | HIS40 | Hydrophobic | Pi-Pi Stacked |
| **Luteolin** | -8.67 | 2.1241 | HIS161 | Hydrogen Bond | Conventional Hydrogen Bond |
|  |  | 2.99673 | TYR22 | Hydrogen Bond | Conventional Hydrogen Bond |
|  |  | 5.14421 | CYS147 | Other | Pi-Sulfur |
|  |  | 4.99554 | HIS40 | Hydrophobic | Pi-Pi T-shaped |
|  |  | 3.83758 | ALA144 | Hydrophobic | Pi-Alkyl |
|  |  | 5.15627 | CYS147 | Hydrophobic | Pi-Alkyl |
| **Daphnetin** | -8.42 | 2.04245 | HIS161 | Hydrogen Bond | Conventional Hydrogen Bond |
|  |  | 2.53471 | GLY166:HN | Hydrogen Bond | Conventional Hydrogen Bond |
|  |  | 2.13996 | THR142 | Hydrogen Bond | Conventional Hydrogen Bond |
|  |  | 5.72744 | CYS147 | Other | Pi-Sulfur |
| **Phlorizin** | -8.31 | 2.13277 | HIS161 | Hydrogen Bond | Conventional Hydrogen Bond |
|  |  | 2.44097 | VAL162 | Hydrogen Bond | Conventional Hydrogen Bond |
|  |  | 2.09524 | THR142 | Hydrogen Bond | Conventional Hydrogen Bond |
|  |  | 3.04341 | GLU24 | Hydrogen Bond | Conventional Hydrogen Bond |
|  |  | 5.875 | CYS147 | Other | Pi-Sulfur |
|  |  | 4.73918 | ALA144 | Hydrophobic | Pi-Alkyl |

**Table S3:** Summary of Comparative Analysis of the Physicochemical Properties, Lipophilicity, Pharmacokinetics, and Drug-Likeness of myricetin

| **SwissADME** | | **Myricetin** |
| --- | --- | --- |
| **Physicochemical Properties** | MW | 318.24 |
|  | #Heavy atoms | 23 |
|  | #Aromatic heavy atoms | 16 |
|  | Fraction Csp3 | 0 |
|  | #Rotatable bonds | 1 |
|  | #H-bond acceptors | 8 |
|  | #H-bond donors | 6 |
|  | MR | 80.06 |
|  | TPSA | 151.59 |
| **Lipophilicity** | iLOGP | 1.08 |
|  | XLOGP3 | 1.18 |
|  | WLOGP | 1.69 |
|  | MLOGP | -1.08 |
|  | Silicos-IT Log P | 1.06 |
|  | Consensus Log P | 0.79 |
| **Pharmacokinetics** | GI absorption | Low |
|  | BBB permeant | No |
|  | Pgp substrate | No |
|  | CYP1A2 inhibitor | Yes |
|  | CYP2C19 inhibitor | No |
|  | CYP2C9 inhibitor | No |
|  | CYP2D6 inhibitor | No |
|  | CYP3A4 inhibitor | Yes |
|  | log Kp (cm/s) | -7.4 |
| **Drug likeness** | Lipinski | Yes; 1 violations |
|  |  | , NHorOH>5 |
|  | Ghose | Yes |
|  | Veber | No; 1 violations |
|  |  | TPSA>140 |
|  | Egan | No; 1 violation |
|  |  | TPSA>131.6 |
|  | Muegge | No; 2 violations |
|  |  | , TPSA>150, H-don>5 |
|  | Bioavailability Score | 0.55 |
